# Risk assessment of Ebola virus disease spreading in Uganda using a two-layer temporal network

**DOI:** 10.1038/s41598-019-52501-1

**Published:** 2019-11-05

**Authors:** Mahbubul H. Riad, Musa Sekamatte, Felix Ocom, Issa Makumbi, Caterina M. Scoglio

**Affiliations:** 1 Department of Electrical and Computer Engineering, Kansas State University, 1701D Platt St., Manhattan, KS 66506 USA; 2 Zoonotic Disease Coordination Office (ZDCO), National One Health Platform (NOHP), Ministry of Health, Kampala, Uganda; 3 World Health Organization Country Office, Kampala, Uganda; 4 Emergency Operations Center, Ministry of Health, Kampala, Uganda

**Keywords:** Computational science, Computational models

## Abstract

Network-based modelling of infectious diseases apply compartmental models on a contact network, which makes the epidemic process crucially dependent on the network structure. For highly contagious diseases such as Ebola virus disease (EVD), interpersonal contact plays the most vital role in human-to-human transmission. Therefore, for accurate representation of EVD spreading, the contact network needs to resemble the reality. Prior research has mainly focused on static networks (only permanent contacts) or activity-driven networks (only temporal contacts) for Ebola spreading. A comprehensive network for EVD spreading should include both these network structures, as there are always some permanent contacts together with temporal contacts. Therefore, we propose a two-layer temporal network for Uganda, which is at risk of an Ebola outbreak from the neighboring Democratic Republic of Congo (DRC) epidemic. The network has a permanent layer representing permanent contacts among individuals within the family level, and a data-driven temporal network for human movements motivated by cattle trade, fish trade, or general communications. We propose a Gillespie algorithm with the susceptible-infected-recovered (SIR) compartmental model to simulate the evolution of EVD spreading as well as to evaluate the risk throughout our network. As an example, we applied our method to a network consisting of 23 districts along different movement routes in Uganda starting from bordering districts of the DRC to Kampala. Simulation results show that some regions are at higher risk of infection, suggesting some focal points for Ebola preparedness and providing direction to inform interventions in the field. Simulation results also show that decreasing physical contact as well as increasing preventive measures result in a reduction of chances to develop an outbreak. Overall, the main contribution of this paper lies in the novel method for risk assessment, which can be more precise with an increasing volume of accurate data for creating the network model.

## Introduction

Ebola virus disease (EVD) is a viral hemorrhagic fever with a high mortality rate^1,2^. The virus is transmitted via direct contact with blood or other bodily fluids of symptomatic individuals; or with contaminated materials such as bedding, clothing, or needles; and direct contact with the dead body of an infected person^2^. People working in healthcare facilities are at high risk of catching EVD. An outbreak of EVD in the eastern Democratic Republic of Congo (DRC) is the largest in the history of the DRC and the second largest recorded outbreak of Ebola ever (after the 2014–16 outbreak in West Africa). As of April 26, 2019, 1,396 Ebola cases have been recorded including 900 deaths and 318 survivors. This is a highly complex Ebola outbreak, which is currently transmitting active transmission in 13 of the 21 affected health zones^3^. Unstable conditions due to armed conflict, outbreaks of violence, and other problems in affected areas complicate public health response activities and increase the risk of disease spread both locally within the DRC and to neighboring countries^3^ such as Uganda, Rwanda, Burundi, Zambia, South Sudan, Central Africa, etc. With the recent outbreak in the DRC, all of these countries are at risk of EVD spreading due to the entrance of someone infected. The DRC outbreak in neighboring districts of Uganda poses a high risk of Ebola introduction. People from the DRC move to Uganda for healthcare, trading, and refuge. Therefore, the public health department has to prepare proper facilities to be prepared for possible EVD outbreak. This requires a risk assessment of EVD spreading dependent on human movement as EVD spreads directly via movement of infected persons and their bodily fluids. Risk assessment will enable public health people to focus their preparedness on building temporary medical facilities, increasing hospital beds, and spreading awareness by taking measures such as avoiding physical contact with infected/unknown people in high-risk areas. In this context, mathematical modeling of infectious diseases can provide information on the progression of EVD as well as effectiveness of different mitigation intervention policies^4–15^.

Past modelling efforts on EVD focused mostly on the deterministic/stochastic compartmental models and agent-based models^6–12,14,15^. Mean-field compartmental models consist of several compartments who evolves in time and describe fraction of the population in each compartment^4,5^. Several mean-field compartmental models has been proposed to describe EVD spreading and investigate impact of interventions^7,8,10–12,14,16,17^. These models are effective in rigorous understanding of EVD spreading. However, they are based on the assumption of homogeneous mixing which is an oversimplification and results in overestimation of cases^18^. Agent-based models used for EVD spreading and risk assessment are very refined models and can be used for practical purposes only with a deep knowledge of human behavior^9,18^.

Network models for infectious disease spreading and risk assessments has opened a new era in management and containment of epidemics^19–21^. A large number of connectivity-driven network models have been proposed for various infectious diseases. These models are particularly well-suited for capturing essential system features where connections among nodes in the network are long lived^13^. A basic assumption with these networks is long-lived contact among individuals, which validates constant links in the network without oversimplification^22^.

Many models (both network and non-network) for EVD spreading have been formulated assuming homogeneous long-lived connections among individuals. Compartmental models have been used in risk assessment, estimating the basic reproductive ratio, and suggesting mitigation measures by fitting transmission dynamics with incidence data^7,9,17,23–27^. However, long-lived connections are not suitable for systems with rapidly changing links^22,28^. Highly contagious/infectious diseases are mostly transmitted from infected to susceptible individuals via physical contact^29^. Therefore, contagious-disease-spreading models are crucially dependent on contact structure among individuals in the network^30^. Considering a network model of human population, the assumption of constant contact with each other is actually an oversimplification of the reality. In general, contact structure among individuals changes with time. These changes in contacts are not completely random; rather, there is always a pattern. For example, a contact pattern will change with changing frequency of occasional or permanent partners for sexually transmitted diseases. Therefore, change of permanent partners can be a long-term process and connectivity-driven network models work fine. However, when we consider the occasional partner change, the connectivity-driven network will fail to capture the frequent change of partners. Several approaches has been proposed for adapting network models with changing contact patterns. One of the earliest concepts is switching networks^5,31^. In this model, the contact network switches among some predetermined networks structures. This model accounts for a changing contact structure with time. However, some predefined structures are required for this network model. In real world, contact structures are highly dynamical and evolve in time^32^. To capture dynamic contact patterns in the network, activity-driven network (ADN) has been proposed^13^. ADNs are very powerful for studying epidemic process when the disease dynamic and contacts evolution shares a common time-scale^33,34^. Activity-driven network provides opportunity to incorporate different real-life scenarios, such as human behavior, movement pattern in the network model^34,35^. ADNs also provide means to model nodes that are likely to have contacts with the rest of the network^13,36^. Flexibility to incorporate different features makes ADN suitable for real-life network models^36,37^. Activity-driven network has been used to capture transmission dynamics of infectious diseases in susceptible-infected-susceptible (SIS) and susceptible-infected-recovered (SIR) model^32,34^.

Activity-driven networks (ADNs) have been widely used for EVD spreading as well. ADNs overcome the simplifying assumption of long-lived and homogeneous contacts among individuals^21,33^. Rizzou *et al*. used an ADN to emulate the dynamics of EVD in Liberia and offer a one-year prediction^21^. The effect on contact tracing on the spreading dynamics has also been quantified using an ADN^38,39^. An activity-driven network has a limitation as it randomly creates new links every time. Therefore, permanent links in the network are not considered in the activity-driven network. In recent time, several modelling frameworks have been proposed to overcome this problem with the ADN by coupling time-varying and static network components. Lie *et al*. proposed a static-activity coupling network^40^, and Nadine *et al*. proposed a framework superimposing an ADN to a static backbone network^41^. These networks integrate persistent contacts with time-varying connections^40,41^. Vajdi *et al*. proposed a two-layer temporal network incorporating both static/permanent links and temporal/occasional links in two different layers^42^.

In this paper, we develop a novel risk assessment framework using a two-layer network with both static/permanent and temporal/occasional links in different layers, our proposed susceptible active-susceptible inactive-infected active-infected inactive-recovered (*S S*_*a*_ *I I*_*a*_ R) model, and the Gillespie algorithm. The two-layer network has a permanent layer reflecting permanent contacts and a temporal layer that incorporates potential contacts. We adapted the Gillespie algorithm with the *S S*_*a*_ *I I*_*a*_ R compartmental model and the two-layer network to see the evolution of disease spread and risk assessment. As an example of the method, we proposed a network for Uganda including 23 districts based on human movement from a focal-bordering Ugandan district to Kampala. In this network, the permanent layer expresses contacts among family members while intra- and inter-district contacts reflect potential contacts due to human movement. Simulation results suggest that making people aware of reduced physical contact while travelling and taking other preventive measures will reduce the number of EVD-infected humans. Results show some districts are more vulnerable to the risk of EVD spreading than others, which may suggest important guidelines for public health personnel in applying interventions and prioritizing resource allocations. Assessed risks are probabilities of infection spreading for our specific scenario based on generic and incomplete movement data, and any change in the network will result in different risks. Therefore, risk assessments in this paper are just some examples of the proposed, novel risk assessment method. The main contribution of this paper is the novel, two-layer temporal network-based simulation tool for risk assessment of EVD spreading, which has the capability to be used for practical purposes when incorporating accurate movement data and model parameters.

The rest of the paper is organized as follows. The risk assessment method section describes the two-layer temporal network, *S S*_*a*_ *I I*_*a*_ R epidemics on the two-layer temporal network, and adaptation of the Gillespie algorithm for risk assessment. Application of risk assessment for Uganda EVD spreading showed an example for two-layer temporal network use in Uganda, simulation results, and discussion. We summarize our conclusions and suggestions drawn from these simulation results in the Conclusions section.

## Risk Assessment Method

In this section we propose a novel method for risk assessment using a two-layer temporal network, *S S*_*a*_ *I I*_*a*_ R spreading model, and the adaptation of the Gillespie algorithm for a temporal network.

### Two-layer temporal network

**Figure 1:**
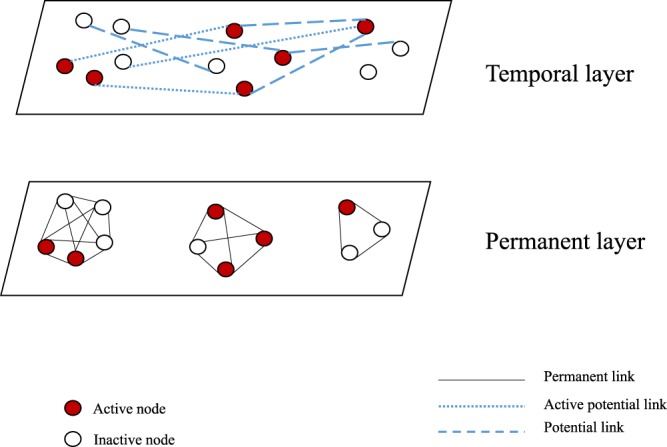
A generalized representation of the temporal network model at a specific time *t* –white circles represent inactive nodes while red circles represent active nodes. Separate rectangles represent two different layers. Dark solid lines show permanent links while dashed lines show links in the potential layer. A potential link becomes active following a Bernoulli distribution with the probability *P*_0_, when both ends of the link are active nodes.

We consider a two-layered network with a population of *N* individuals. In the two-layered model, individuals can have links among them in both layers. The intersection of the two network layers are assumed empty. We denote layers in the network as *L*_1_ and *L*_2_. In the first layer, *L*_1_, links among individuals are considered permanent. Links in the second layer, *L*_2_, are considered as potential links. In the subsequent section of this work, we call *L*_1_ a permanent layer while *L*_2_ is called a temporal layer^42^. Therefore, links in the two layers will be referred to as permanent and potential/temporal links, respectively. Links in both layers are established based on some certain probability distributions. Permanent links are always active in the network while potential links are activated with a probability only when individuals at both ends of the link are active simultaneously. Activation of individuals are driven by an activity-firing rate (*γ*) as discussed in the ADN. In stochastic realization of the network, once both nodes are active, the link between them becomes active with a Bernoulli distribution having the probability *P*_0_. The generalized structure of the temporal network is presented in Fig. 1.

In the permanent layer, a link can always transmit infection whether it is active or not. These permanent links are always present in the network. A link in the potential layer can transmit infection only when it connects two active individuals. If there is a link between two nodes in the temporal layer, that link might be active or not, depending on the status of the nodes. The potential link becomes active with a probability *P*_0_ only when both nodes are active simultaneously. When one node in the active link becomes deactivated, the link vanishes. Therefore, probability of infection transmission through the link becomes zero again. The process of a node becoming active or inactive is assumed as a Poisson process with a rate of *γ*. A node will stay active for an exponentially distributed period with an expected value of *γ*^−1^. Thus we can assume a high value of *γ* for a specific node will reflect the reduced duration of an active potential link. The parameter *γ* is referred to as the activity rate in the subsequent sections of the paper. Increased *γ* means an increasing frequency of a node changing its status i.e., becoming active/inactive. For example, we assume an individual becomes active once he starts a movement/trip and stays active until the trip is finished. Therefore, decreasing *γ* means an increasing length of the trip and a decreasing frequency of this kind of trip. Moreover, if a node does not participate in the occasional contacts, it never becomes active and *γ* is set to equal zero for that node. Temporal link disappears with the when either of nodes in the link deactivates^42^. This temporal network is different from the widely used, activity-driven network in the contact structure among individuals. In contrast with the activity-driven network, there are permanent links, along with different temporal links, in the proposed two-layer temporal network.

### *SS*_*a*_*II*_*a*_*R* epidemics on two-layer temporal network

In this section, we describe the modification of the susceptible-infected-removed (SIR) model for our two-layer temporal network. The SIR model is a popular approach for studying infection spreading where infected people die, or eventually recover and gain life-long immunity. For diseases such as chicken pox, EVD, etc., the SIR model has the potential to describe the disease dynamics and infection spreading. In the SIR model, each individual is either susceptible, infected, or removed/recovered. We assume infection and recovery processes are independent Poisson processes. A susceptible node catches the EVD infection from an infected person, and this transition happens with transmission rate *β*. Once a person becomes infected, he/she stays infected for a certain period, namely an infectious period. After the infectious period, individuals recover or are removed from the infection. The rate at which an infected person leaves the infected state is called recovery/removal rate, which is the inverse of the infection duration. The recovery/removal rate is denoted as *δ*, which has a unit *time*^−1^. Up to this point, we have discussed the basic SIR model. However, for our two-layer temporal network, we must also consider status of the individual (active/inactive). Combining our temporal network model and SIR spreading process, we have a total of six states an individual can occupy. However, the recovered/removed population does not participate in disease transmission. Therefore, their status does not have any impact on the disease dynamics, and we can combine active and inactive recovered/removed compartments. Therefore, the model can be expressed as a five-compartment *S S*_*a*_ *I I*_*a*_ R model, where compartments are inactive susceptible, active susceptible, inactive infected, inactive infected, and recovered.

If the probability of node *i* occupying inactive susceptible, active susceptible, inactive infected, active infected, and recovered in the stochastic spreading process is expressed as *S*, *S*_*a*_, *I*, *I*_*a*_, and *R*, respectively, then equations for the time evolution can be expressed as follows:

1 $${S}^{i^{\prime} }=-\,{\gamma }_{1}^{i}{S}^{i}+{\gamma }_{2}^{i}{S}_{a}^{i}-\beta \sum _{j}\,{a}_{1}^{ij}{S}^{i}({I}_{i}^{j}+{I}_{a}^{j})$$

2 $${S}_{a}^{i^{\prime} }={\gamma }_{1}^{i}{S}^{i}-{\gamma }_{2}^{i}{S}_{a}^{i}-\beta \sum _{j}\,{a}_{1}^{ij}{S}_{a}^{i}({I}^{j}+{I}_{a}^{j})-\beta \sum _{j}\,{X}_{0}^{ij}{a}_{2}^{ij}{S}_{a}^{i}{I}_{a}^{j}$$

3 $${I}^{i^{\prime} }=-\,{\gamma }_{1}^{i}{I}^{i}+{\gamma }_{2}^{i}{I}_{a}^{i}+\beta \sum _{j}\,{a}_{1}^{ij}{S}_{a}^{i}({I}^{j}+{I}_{a}^{j})-\delta {I}^{i}$$

4 $${I}_{a}^{i^{\prime} }={\gamma }_{1}^{i}{I}^{i}-{\gamma }_{2}^{i}{I}_{a}^{i}+\beta \sum _{j}\,{a}_{1}^{ij}{S}_{a}^{i}({I}^{j}+{I}_{a}^{j})+\beta \sum _{j}\,{X}_{0}^{ij}{a}_{2}^{ij}{S}_{a}^{i}{I}_{a}^{j}-\delta {I}_{a}^{i}$$

5 $${R}^{i^{\prime} }=\delta {I}_{a}^{i}+\delta {I}^{i}$$

**Figure 2:**
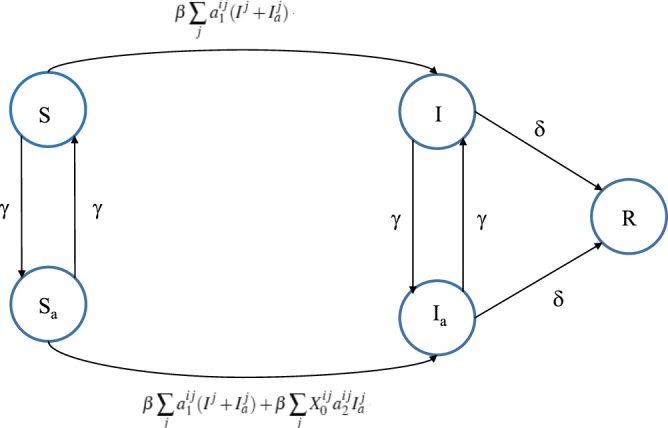
Node-transition diagram for exact/stochastic spreading process used in this work – each circle represents a compartment whose name is written inside the circle. Expressions written over directional lines show probabilities of transition from origin to destination circle (compartment).

${a}_{k}^{ij}$ is the element of adjacency matrix *A*_*k*_, where *k* = 1 for the permanent layer and *k* = 2 for the potential layer. Equations (1–5) express stochastic equations for *S S*_*a*_ *I I*_*a*_ R spreading. The variable ${X}_{0}^{ij}$ is a Bernoulli random variable that has a value of one with a probability of *P*_0_. This random variable is drawn each time a pair of active nodes *i*, *j* with a potential link between them occurs, regardless of their disease status. It can be translated to a real-life expression as follows: when both nodes in a potential link are active, the link can be active with a probability of *P*_0_. We have presented an elaborate diagram illustrating how the parameters in the exact equation drive the state transitions and state occupancy probability in Fig. 2.

Therefore, the model used in this work is a stochastic model and the Gillespie algorithm was adapted accordingly for this exact/stochastic process.

### Adaptation of gillespie algorithm

Gillespie algorithm has been widely used to simulate stochastic processes for static network (permanent contacts)^43–47^ and dynamic/time-varying networks (temporal/potential contacts)^42,48^. However, our two-layer temporal network has both static and temporal contacts. Therefore, Gillespie algorithm was adapted to the changed network state at every time step retaining permanent contacts.

- Initialize the number nodes in the network (*N*), state transition matrices, maximum number of events and the final time for simulation. Find the transition probability *R*_*i*_ for each node at the next time step and ${R}_{tot}={\sum }_{j}\,{R}_{j}$, where *j* = 1, 2, ...*N* and keep track of the status of the nodes (active/inactive)
- Find the time for the next event which is exponentially distributed with a rate *R*_*tot*_. The second step is to select a node according to the probability distribution *Pr*(*i*) = *R*_*i*_/*R*_*tot*_ which will make a state transition. Later we select a state where the transition will happen.
- Increase the time by time calculated in Step 2. The main difference lies in the update of transition rates for each nodes in the network once a transition occurs for our adaptation of Gillespie algorithm. Nodes can change their status (active/inactive) as well as changing their states (susceptible, infected, recovered). Therefore, every time a transition occurs, the status of the nodes needs to updated and recorded. When a node is in the permanent layer, it’s transition probability has the impact of both layer’s neighbours. However, when the node is in temporal layer, it only has the impact of its neighbours on the temporal layer. Therefore, the algorithm is modified to account for the network with two different layer’s impact on the transition probabilities.
- Go back to Step 2 unless the stop condition is reached (maximum number of events, the final time for simulation, or *R*_*tot*_ < *Tolerance*).

### Calculation of risk

As the spreading process in our two-layer temporal network was highly stochastic, we performed two hundred simulations for each combination of parameters. We kept track of each node’s status and counted the numbers of simulations in which a particular node was infected. This count was later used to calculate the risk of EVD spreading in each district. The formula to calculate spreading risk to a specific district is presented in Eq. (6).

6 $$Ris{k}_{j}=\frac{\mathop{\sum }\limits_{n=1}^{{N}_{j}}\,{I}_{n}}{{N}_{j}{N}_{simulation}}$$

where, *Risk*_*j*_ = Ebola spreading risk of district *j*, *I*_*n*_ = Number of simulations where node *n* is infected, *N*_*simulation*_ = Number of simulations, *N*_*j*_ = Total number of population in *j*^*th*^ district, and *j* = 1, 2, 3, ...., 23.

Once we calculate the risk, we can use any mapping software such as ArcGIS software to create risk maps. Risk maps provide a visual representation of of spreading risks.

### Calculation of confidence interval

Our simulation framework is an event-based algorithm, which randomly chooses the time when the next event will occur and what that event will be. Therefore, these events are not homogeneously time slotted. We perform a time regularization, where the temporal window for simulation is divided in equal periods and the number of events are calculated within each period. We obtained the size/number of individuals in each compartment from events in each period. We performed 200 simulations for each parameter set and recorded number of individuals in each compartment for all periods. Later, we found the range within which 95% of the simulation results fall in each period.

### Application of risk assessment for Uganda EVD spreading

We applied our risk assessment method for EVD in Uganda using a generalized movement pattern and some specific model parameters. First, we formulated the two-layer temporal network for Uganda. Later we used the network with the *S S*_*a*_ *I I*_*a*_ R model and the modified Gillespie algorithm to track the number of infected humans after a certain period and assessed the risk of EVD spreading to different spatial locations. Simulation results and discussion for specific scenarios are presented later in this section.

### Two-layer temporal network for Uganda

A recent Ebola outbreak in the DRC created a possibility of EVD infection in Uganda. We proposed a two-layer temporal network for EVD spreading in Uganda. We have observed the focus of Ebola preparedness by the ministry of health and partners to select a possible point of entry for an Ebola patient from the DRC. We found the Kasese district was at high risk of an EVD- infected person’s entry point. Therefore, we chose this district as our point of entry. Once an infected person from the DRC entered into Uganda through the bordering districts, he or she met susceptible people in that location. EVD-infected persons, being highly contagious, can spread the infection to people they meet. People move from one location to another for different reasons and there is always a pattern for this movement. We created a network based on people’s movement for different purposes such as fish trade, cattle trade, and general movement. The movement pattern and districts in the movement paths were obtained from confidential data provided by the ministry of health in Uganda. We used these data to formulate an example of human movement network for some selected districts in Uganda. Human movement from one location to another is largely motivated by three different purposes in Uganda:

1. Fish traders move in a southward direction from the point of entry.
2. General movement for shopping, visiting relatives, search of work, or travelling for various purposes starts at the point of entry and go all the way to the capital city of Kampala. People mostly travel from rural areas to neighboring big cities. They meet other people there and this results in a certain mixing among individuals from different locations. This mixing happens throughout this movement path. Several of these movement paths are presented in Fig. 3 with green arrows.

**Figure 3:**
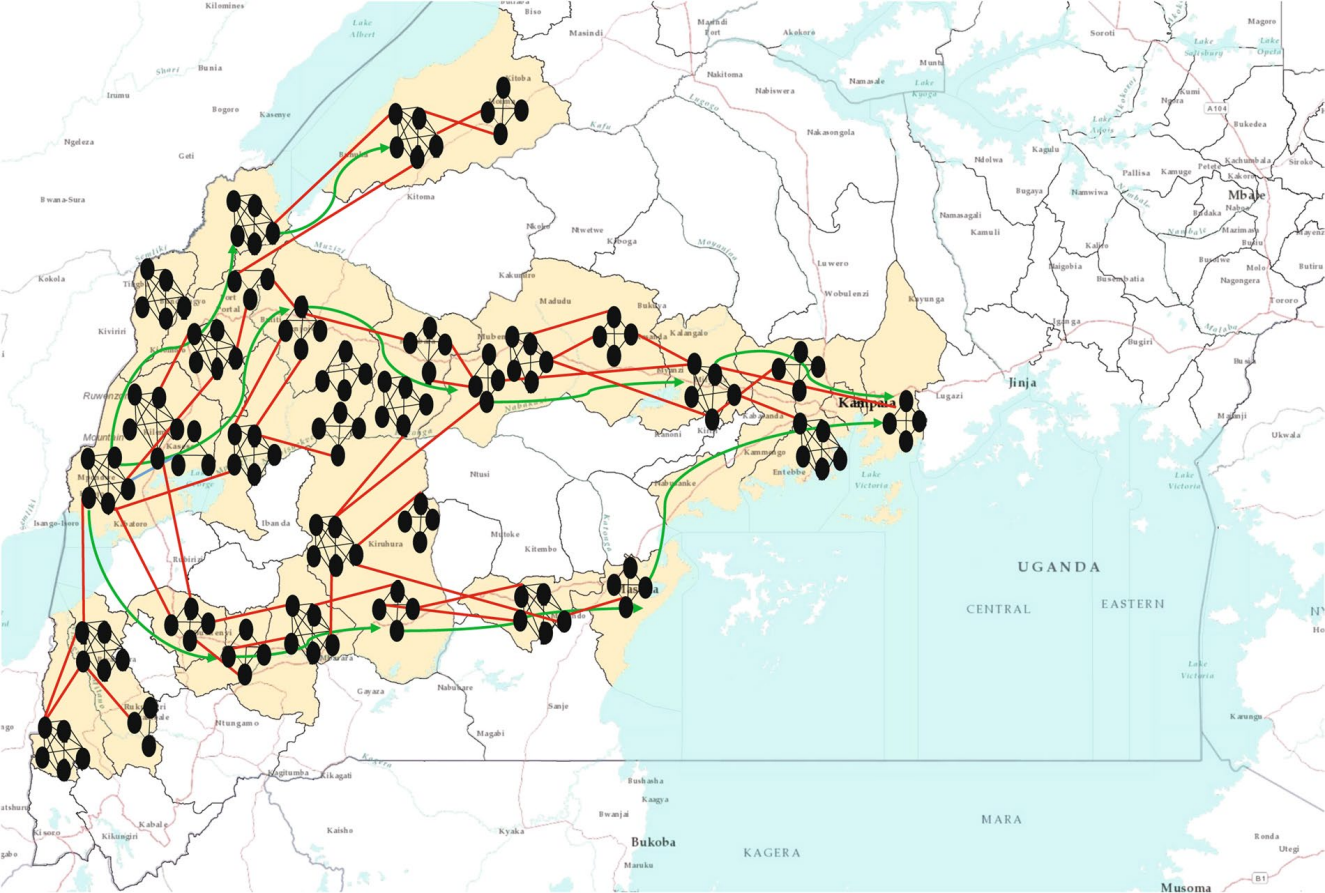
Two-layer temporal network for EVD spreading in Uganda. The districts considered for our network model are coloured in baize (23 districts). Small black oval shapes represent individual human beings. This figure does not represent the actual network, rather it is a visual representation of the two-layer network. Each cluster of human represents a households and black lines among them represents permanents contacts. Red lines represent contacts in potential layer. These links only become active when both individuals in the link are active simultaneously. A link represents a possibility of pathogen transmission. Green directional arrow represents the directions of human movements.
3. Limited movements due to cattle trade are mostly local or between neighboring districts. Long-distance cattle trade happens at the commercial scale and does not include much movement of people as they move mostly via organized transportation systems.

We created a specific network for human movement from Kasese to Kampala based on general movement information. This network was used to show an example of the method developed for risk assessment. As we described in our temporal network section, we assumed two layers in the network. The permanent layer consisted of contacts of each individual within each household. Each family in Uganda has an average of six children^49^. Including parents, we roughly assumed around eight people within each household. Therefore, we assumed a population distribution where each household had five to ten members.

Within each household, we assumed permanent links among family members. Therefore, links within each household constructed the permanent layer in the Uganda EVD spreading network. The potential layer was formed by incorporating previously discussed human movement. An individual becomes active once he or she is in movement and stays active until he or she finishes the movement. Once a node is in movement (active), its potential link can be activated if the other node in the link is active as well. This can be explained as follows: node *i* has potential links with a set of nodes named *J* throughout the whole network. Therefore, once node *i* is active, potential links with any of the active nodes in *J* can be activated following a certain probability. This link-activation structure is crucial in proper representation of the contact structure. Two moving nodes (active) can meet each other in different places such as transportation, marketplace, visiting sites, etc. Usually the movement pattern between individuals follows a general structure. Within each location, a probability exists that individuals encounter each other for various purposes. Inter-location contacts also follow some structure rather than being completely randomized. People usually flock into big cities or towns nearby where they encounter local active people or others coming to that location. When this happens, if two of these active individuals have a potential link, that link is activated with a certain probability distribution (Bernoulli distribution with probability *P*_0_).

**Figure 4:**
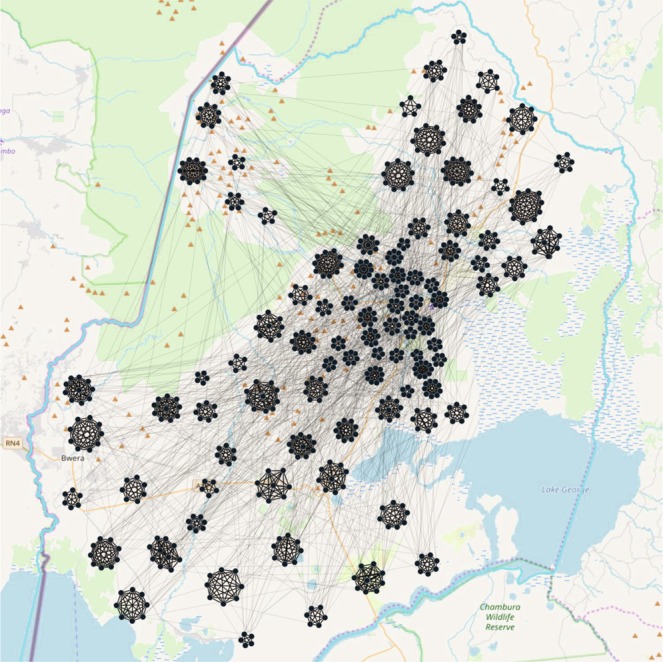
Zoomed view of a district in EVD spreading network in Uganda. We chose Kasese district for this visualization. However, population in the specific district is scaled down for a comprehensible representation of the network. Small clusters represent individual households (permanent layer) and lines between clusters represent temporal links.We created the network using Gephi-0.9.2 (https://gephi.org/) and the map using ArcMap 10 (http://desktop.arcgis.com/en/arcmap/). We have used Inkscape 0.92.3 (https://inkscape.org/) to superimpose the map and network together.

A zoomed up view of a portion of the network (Kasese district) is presented in Fig. 4. This shows a scaled down version of the actual network for visualization purpose.

Districts used in our Uganda network are presented in Table 1. We used the centroid of each district to find distances between districts while formulating the network. However, this is one realization of the network built using available movement data. Some Ugandan districts are not included in our assessment; therefore, there may have been districts at high risk that were excluded from the risk assessment performed in this study. We scaled the population of each district in our network by 1,000 for computational purposes. As our main purpose was to evaluate the risk of EVD, this scaling greatly reduced the computational complexity. We have incorporated previously discussed movement patterns along different paths from one point of entry to Kampala in the network. These movements and their directions are also shown in Fig. 3. We considered all districts that were in movement paths of any nature toward Kampala from Kasese. Although all bordering districts were at risk of Ebola introduction, we focused on demonstrating the application of our method when the initial infections were in the Kasese district.

**Table 1:** Districts considered in our Uganda two-layer temporal network.

Districts	Population
Bundibugyo	224387
Bunyangabu	181200
Bushenyi	234440
Hoima	572986
Kabarole	469236
Kampala	1507080
Kamwenge	414454
Kanungu	252144
Kassanda	100038
Kasese	694992
Kyegegwa	159800
Kyenjojo	422204
Lwengo	267300
Masaka	297004
Mbarara	472629
Mityana	328964
Mpigi	250548
Mubende	684337
Mukono	594804
Ntorko	70900
Rukungiri	314694
Sheema	180200
Wakiso	1997418

In summary, our network for Ebola spreading in Uganda consisted of 23 districts where the permanent layer incorporated contacts among individuals within a household. The potential layer reflected contacts between individuals when both ends of the link were active during movement and had a possibility of pathogen transfer between them.

### Simulation setup

Upon formulation of the two-layer network, we performed simulations with the *S S*_*a*_ *I I*_*a*_ *R* model for EVD spread using the Gillespie algorithm^42,50^. We conducted our simulations with two major goals – to observe the progression of EVD spreading over time throughout the network and evaluate the risk of spatial spreading for this specific scenario. In our simulations, we had four different parameters. They were the rate at which individuals become active and inactive, generally called activity rate *γ*; the transmission rate *β*; the recovery rate *δ*; and the probability *P*_0_ of an active link between two simultaneously active individuals in the potential layer. These values are hard to estimate and due to high stochasticity in the people’s movement pattern, movement parameters, i.e., (*γ*), cannot be expressed with a single value. The transmission rate *β* is also variable and takes different values for different outbreaks depending on the contact pattern among individuals. Therefore, we performed simulations using multiple values of each parameter to explore the sensitivity of epidemic size and spreading risk. For each parameter set, we calculated the total number of infected individuals with time and created risk maps.

We presented the number of infected individuals in each time step for each parameter set. As we had multiple parameters, we chose the value of *P*_0_ = 0.7 and 0.1, which represented a 70% and 10% chance of an active potential link between two active nodes. We chose *γ* = 0.1 and 0.5 for the activation/deactivation rate (activity rate) and ${\gamma }^{-1}$ is the average time an individual is active. The value of *γ* has the following real-life explanation: a particular individual becomes active in every ${\gamma }^{-1}$ days and once active, stays active for the next ${\gamma }^{-1}$ days. For example, *γ* = 0.1 represents the frequency of a node changing its state (inactive to active, active to inactive) every 10 days.

## Results and Discussion

We initiated our simulation with a single, active infected person in the Kasese district and tracked the status of each node in the network for a period of 150 days to see how each node was changing its status (inactive susceptible, active susceptible, inactive infected, active infected, and recovered). At the end of the simulation time (150 days), the total number of infected people in the outbreak were calculated and the risk of a specific node being infected during the outbreak was assessed.

To measure the progression and severity of EVD spreading, we tracked total number of infected people and a cumulative number of infected people for a period of 150 days. For any infection spreading, some important measures are size of the peak infection (maximum number of simultaneously infected individuals), time to reach that peak, and total number of infected within an outbreak (epidemic size)^47^. Therefore, we designed our simulation for tracking both the number and cumulative number of infected at each time step. Our simulation results are presented in the subsequent parts of this section. We had chosen values of transmission rate *β* to explore a varying range of transmission potential given contact with infected individuals. We have calculated the 95% confidence interval from 200 simulations for number of human in each compartment at each period and final epidemic size. Simulation results with confidence interval are shown in Supporting material. For easier demonstration and comparison, we have presented simulation results without confidence interval in the Results section.

**Figure 5:**
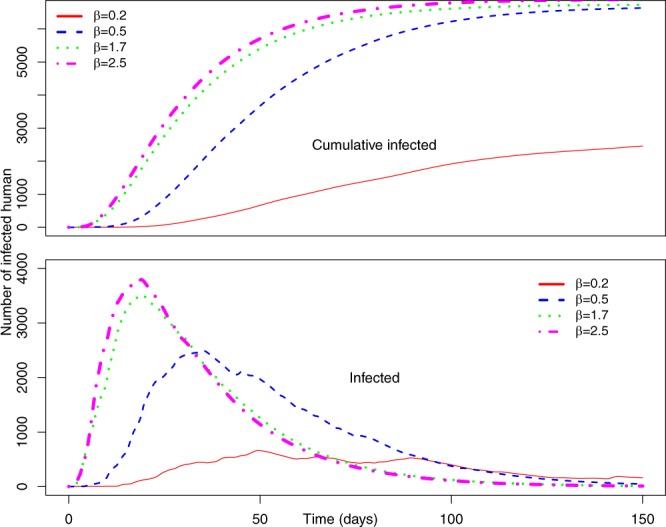
(Top) Average number of cumulative infected and (bottom) average number of infected humans in the Uganda Ebola network for *P*_0_ = 0.7, *γ* = 0.5 –four different colors represent the average number of infected humans for four values of *β*. Red, blue, green, and magenta lines correspond to the number of infected humans for *β* = 0.2, 0.5, 1.7, and 2.5, respectively.

Figure 5 represents the number of cumulative infected and infected humans for *P*_0_ = 0.7 and *γ* = 0.5, and a varying range of transmission rate *β*.

Within Fig. 5, the top graph represents the average cumulative number of infected while the bottom plot represents the average number of simultaneously infected humans at different time steps.

From Fig. 5 (*P*_0_ = 0.7 and *γ* = 0.5), it is evident that with the increase of *β*, infection size increases rapidly. An increase of *β* from 0.2 to 0.5 increases infection size from 2,459 to 6,634. Therefore, a very small increase in transmission causes a huge increase in the total number people infected with EVD when we assumed a 70% chance of pathogen transmission once an active infected individual comes in contact with another active susceptible person in the potential layer. In addition, in this simulation set, the potential layer is assumed to be highly active/mobile as people are assumed to become active and inactive within an average period of two days.

The number of simultaneously infected people at a certain time is very important for public health personnel^51^. More infected people means increased preparation of hospital beds, doctors, and medical supplies^52^. Therefore, once an outbreak occurs, it is very important to have an idea about the maximum number of simultaneously infected people (size of the peak infection)^47^. In Fig. 5, the bottom plot shows simultaneously infected people at each time step. In the plot, peak infection size also increases with *β*. However, when *β* = 0.2, the peak is not very pronounced and peak infection size is around 600. However, for other values of *β*, the peak is more pronounced and peak infection size is more than 2,500 for all other values of *β*. The time to reach the peak for *β* = 0.2 is around 50 days. With the increase of *β*, the infection plot becomes skewed to the left, meaning faster arrival at peak infection size. Faster arrival to peak infection and greater value of peak infection size indicates a widespread epidemic outbreak. Therefore, simulations for this specific parameter set indicate a widespread and severe outbreak for *β* > 0.5 where more than 50% of our total population in the network becomes infected.

For each parameter set, we had estimated the risk of EVD spreading to different spatial locations from a single infected individual at the Kasese district. We had created risk maps using ArcGIS for EVD spread to distant locations. We had used an equation for risk explained previously to calculate the risk. Values of risk are classified in five different categories as presented in Table 2.

**Table 2:** Classification of risk for our spatial locations based on the value of risk parameter.

Risk	Value of risk parameter
No risk or not considered	0
Low risk	0 < risk ≤ 0.2
Moderate risk	0.2 < risk ≤ 0.4
Medium risk	0.4 < risk ≤ 0.6
High risk	>0.6

**Figure 6:**
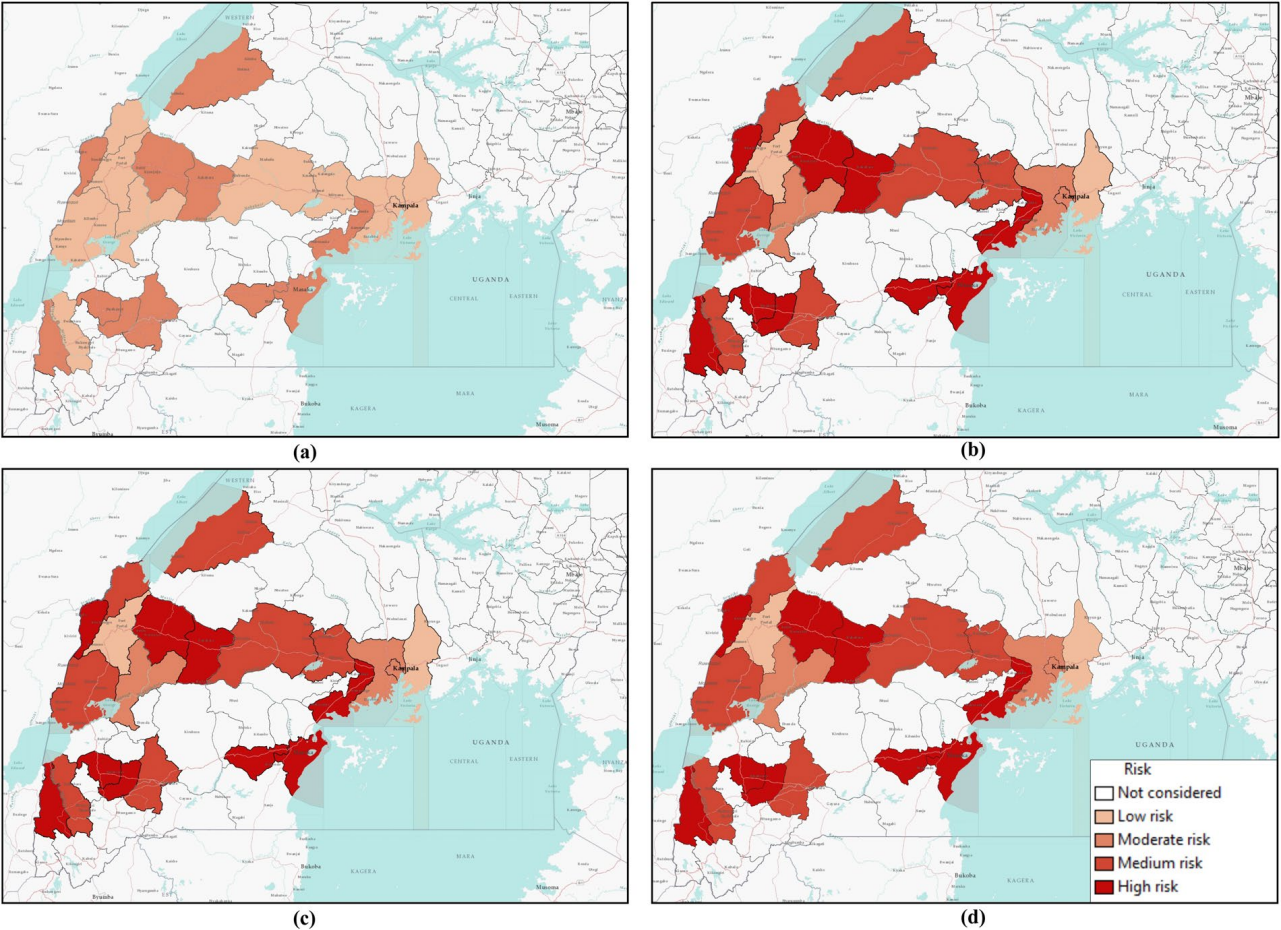
Risk map of Ebola spreading within selected 23 districts in Uganda for *P*_0_ = 0.7, *γ* = 0.5, and (**a**) *β* = 0.2, (**b**) *β* = 0.5, (**c**) *β* = 1.7, (**d**) *β* = 2.5. The map is colour coded according to the risk of Ebola spreading.

Risk maps in Fig. 6 show all neighboring districts in the southern part of Uganda, although we have considered 23 districts. Therefore, some districts on the maps had not been considered in our two-layer temporal network and, hence, have not been assessed for risk.

Figure 6 shows a risk map for four selected values of *β* and *P*_0_ = 0.7, and *γ* = 0.5. The map is colored with a monochromatic color gradient, which increases with increased risk. Therefore, districts that are white on the map are not considered in the network model.

From the risk map for *β* = 0.2 in Fig. 6, we can see that all of our selected locations are at low or moderate risk as the transmission rate is very low. However, some districts are at comparatively higher risk than others are. For this specific scenario with *β* = 0.2, Bundibugyo, Bushenyi, Kyegegwa, Kyenjojo, Masaka, Mpigi, and Sheema districts are at higher risk than other districts in our network. However, the aforementioned districts are at moderate risk of Ebola spreading while other districts are at low risk for *β* = 0.2. Increasing the value of *β* increases risks proportionately, which can be seen from Fig. 6. Color gradients increase in risk maps with increasing *β*. In the risk maps for other values of *β*, a similarity in risk is observed. This is evident from similar color gradients of districts on all three maps for *β* = 0.5, 1.7, and 2.5, which can be explained from similar epidemic size and comparable infection peaks for these values of *β* (Fig. 5). Therefore, we have similar infection spreading in these cases and assessed risks are similar (not same, but they are in the same interval as presented in Table 2). There was an increasing trend in the value of calculated risk parameters with the increase of *β*, although on the map they are included in the same interval. Districts in the temporal network for *β* = 0.5, 1.7 and 2.5 with assessed risks are presented in Table 3.

**Table 3:** Districts in the network and their associated risks for EVD spreading.

Risk	Districts
High risk	Bundibugyo, Bunyangabu, Bushenyi, Kanungu, Kyegegwa, Kyenjojo, Lwengo, Masaka, Mpigi, Sheema
Medium Risk	Hoima, Kasese, Mbarara, Mityana, Mubende, Ntoroko, Rukungiri, Kassanda
Moderate risk	Kampala, Kamwenge, Wakiso
Low risk	Kabarole, Mukono

Table 3 shows that 10 of 23 districts are at high risk of EVD spreading during an outbreak. With a current outbreak in the DRC, it is expected that bordering districts will be at high risk of EVD spreading. However, our simulation results have the capability to incorporate movement data that shows non-bordering districts can also be at high risk due to infected persons’ movement. This is can be easily seen from Fig. 6, where some non-bordering districts demonstrate high or medium risk of EVD spreading.

**Figure 7:**
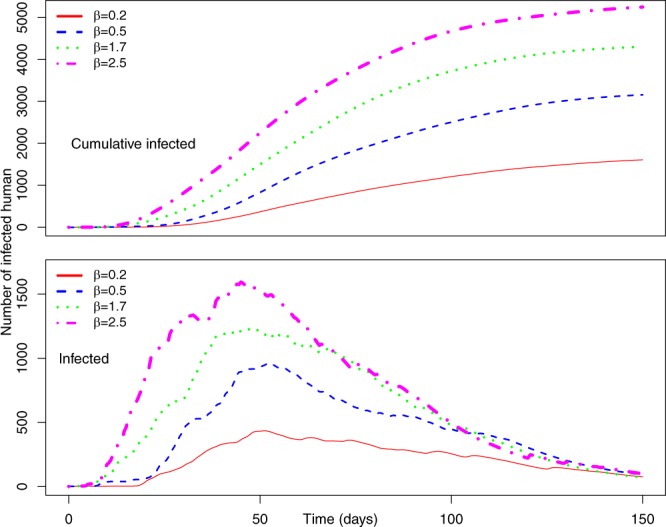
(Top) Average number of cumulative infected and (bottom) average number of infected humans in the Uganda Ebola network for *P*_0_ = 0.7, *γ* = 0.1 – four different colors represent the average number of infected humans for four values of *β*. Red, blue, green, and magenta lines correspond to the number of infected humans for *β* = 0.2, 0.5, 1.7, and 2.5, respectively.

Figure 7 represents the number of infected individuals for *P*_0_ = 0.7 and *γ* = 0.1. *γ* = 0.1 means individuals have the probability of becoming active and inactive with an exponentially distributed time, which has an average of 10 days. Therefore, decreasing *γ* decreases the probability of a human becoming active, while increasing the time of him/her staying active. Peak infection increases from 450 to 1,700 for our selected values of *β* = 0.2 to 2.5, while the cumulative number of infected increases from 59 to 1,607.

**Figure 8:**
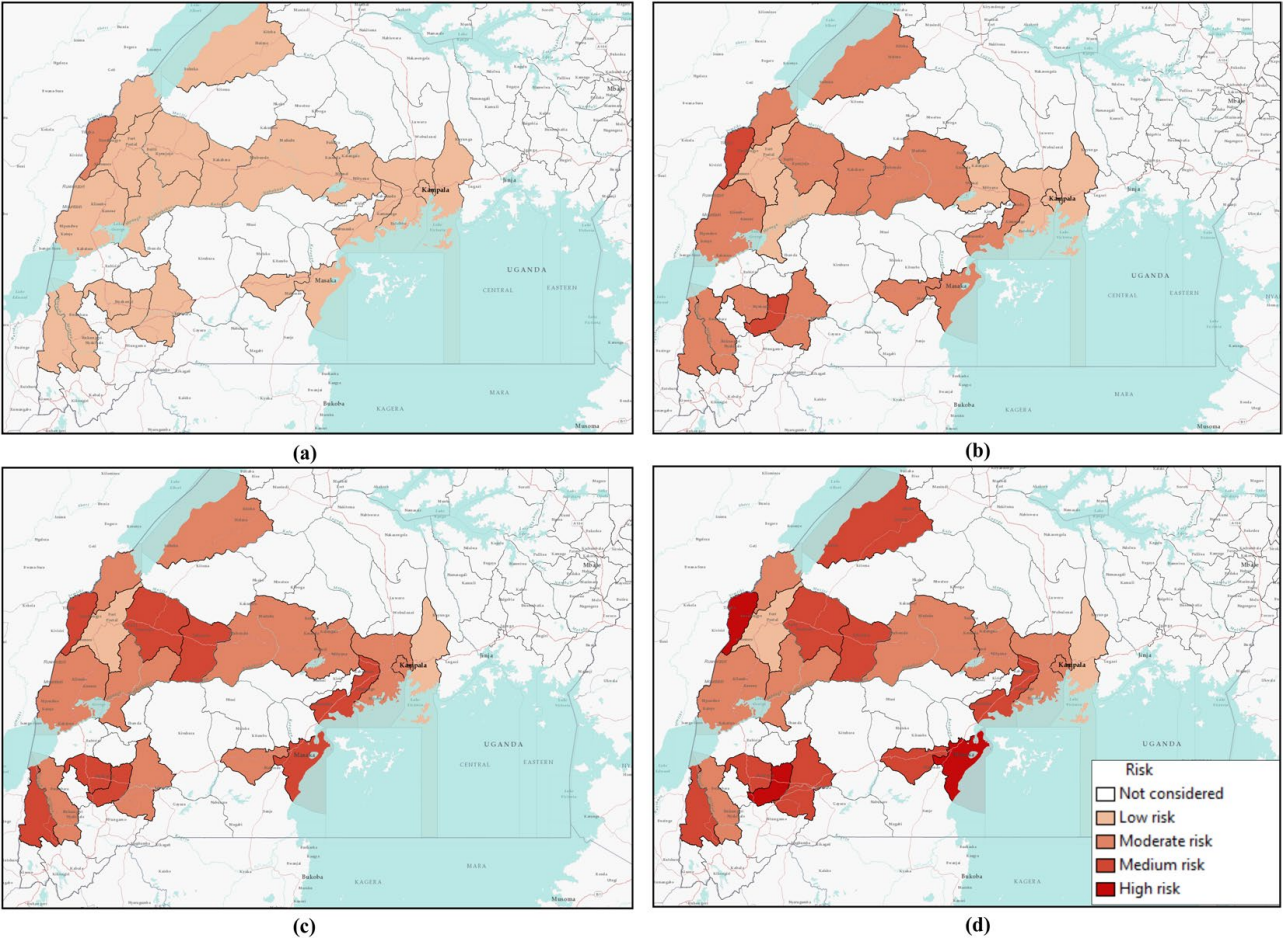
Risk map of Ebola spreading within selected 23 districts in Uganda for *P*_0_ = 0.7, *γ* = 0.1, and (**a**) *β* = 0.2, (**b**) *β* = 0.5, (**c**) *β* = 1.7, (**d**) *β* = 2.5. The map is colour coded according to the risk of Ebola spreading.

Comparing simulation results presented in Figs 5 and 7 for similar values of *β*, epidemic size as well as peak infection is always higher for higher values of *γ*. Higher values of *γ* can be translated to increased human movement in our network. As we are assuming an active person is a travelling person, a higher *γ* for a person means frequent short trips, while lower *γ* means infrequent longer trips. Higher *γ* increases the time a human will be in the active (mobile) state, while a lower value indicates longer length of an individual staying active^42^. Therefore, it is convenient to assume that while an infected individual is active for a longer period of time, this will eventually spread the infection to more active individuals in the potential layer^53^. However, our simulation results show otherwise. An increase in the value of *γ* increases infection size as well as peak infection size (Figs 5 and 7). Therefore, our simulation results show the frequency at which individuals become active or inactive dominates over the time span of the individual staying active. This is evident from the higher size of the epidemic from the higher value of *γ*. For *β* = 0.2, the cumulative number of infected after 1,607 is when it is 59 and we have *γ* = 0.5. The value of the cumulative number of infected is also higher for other cases as well. The infection reaches its peak slowly (approximately 50 days) irrespective of the value of the transmission rate. Therefore, lower value of the activity rate *γ* means a lesser number of simultaneously infected people and a slower spread of infection within the spatial locations (Figs 6 and 8). As we discussed earlier, a lower value of *γ* reflects the reduced movement of people. From comparisons between simulation results in Figs 5 and 7, it is evident that human movement is critical in the severity and speed of EVD spreading. As frequent human movement (frequent short trips) spreads EVD very quickly, reduced human movement may minimize the severity of the EVD spread^54^. This is also evident from the risk map shown in Fig. 8 for reduced *γ*. From the map, we can see that Bundibugiyo, Sheema, and Masaka are three districts most at risk of Ebola spreading for our specific network model. For these three districts, the risk of spreading is comparatively higher than other districts, even when the value of the transmission rate is very low. For example, these districts are in a moderate-risk zone while others are in a low-risk zone for *β* = 0.2. However, with the increase of *β*, the value increases and for our highest value of *β* = 2.5, these three districts are at a high risk of Ebola spreading. Table 3 presents districts in the temporal network for *β* = 2.5 with assessed risks.

During the network creation, some districts were assumed possible mixing places, which makes them more vulnerable than others. Therefore, simulation results and evaluated risks were obtained according to the network structure. An example of dependency on the network structure is evident from Figs 6 and 8, showing the district of Kabarole at low risk for all values of *β*, despite being a bordering district to DRC. This low risk for Kabarole reflects the fact that this district was not considered as a mixing place in our network. This demonstrates our method’s adaptability to specific data about each location in the network.

Comparing Tables 3 and 4, it is evident that when we have a lower *γ*, risk decreases significantly. For the same values of *β*, lower *γ* reduces the risk of Ebola spreading. When *β* = 0.5 and *γ* = 0.5, there are 11 high-risk districts. Decreasing the value of *γ* to 0.1 results in only three high-risk districts for *β* = 2.5. For other values of *β* and *γ* = 0.1, none of the districts in our network model are at high risk. Therefore, reducing human movement has shown a significant decrease in EVD spreading. However, reducing human movement is not practical, as it cannot be controlled. Therefore, we focus on the parameter *P*_0_, which express the possibility of pathogen transfer via an active potential link. *P*_0_ can be expressed as the probability of an active infected person spreading the virus to an active susceptible person. The value of this parameter is dependent on direct physical contact between the infected and the susceptible. In our previous set of simulations, we used *P*_0_ = 0.7. Now, if we can decrease the possibility of physical contact among humans in case of an outbreak, it would be equivalent to a reduced possibility of virus transfer. To observe the impact of reduced physical contact i.e., lower *P*_0_, we conducted a simulation for a *P*_0_ = 0.1 reflecting only a 10% possibility of pathogen transfer via an active link while one of the humans is infected.

**Table 4:** Districts in the network and their associated risks for *P*_0_ = 0.7, *γ* = 0.1 and *β* = 2.5.

Risk	Districts
High risk	Bundibugyo, Masaka, Sheema
Medium Risk	Bunyangabu, Bushenyi, Hoima, Mbarara, Mityana, Mpigi, Kyegegwa, Kyenjojo, Lwengo, Kanungu
Moderate risk	Kampala, Kamwenge, Wakiso, Rukungiri, Ntoroko, Mubende, Mityana, Kasese, Kassanda
Low risk	Kabarole, Mukono

Decreasing the probability of EVD spreading (i.e. *P*_0_) to 10% from 70% via a contact in a potential layer significantly reduces infection size as well as numbers of simultaneously infected humans. However, while changing the *P*_0_, similar values of *γ* are used as before.

**Figure 9:**
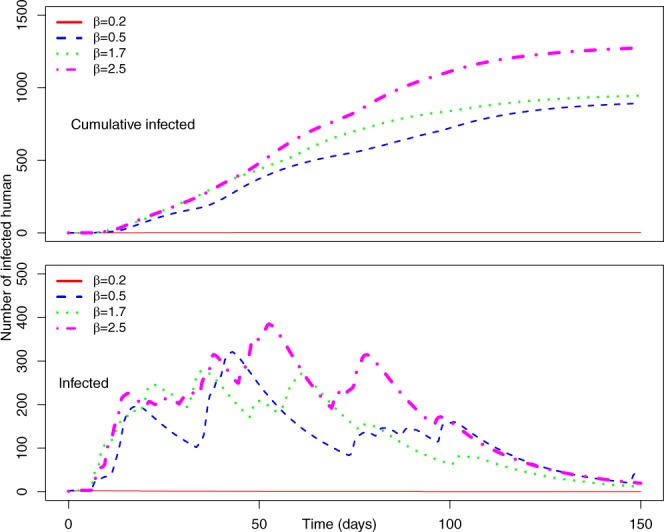
(Top) Average number of cumulative infected and (bottom) average number of infected humans in the Uganda Ebola network for *P*_0_ = 0.1, *γ* = 0.5 –four different colors represent the average number of infected humans for four values of *β*. Red, blue, green, and magenta lines correspond to the number of infected humans for *β* = 0.2, 0.5, 1.7, and 2.5, respectively.

Figure 9 shows cumulative infected humans for *γ* = 0.5 and *P*_0_ = 0.1. For our lowest value of *β* = 0.2, the cumulative number of infected humans is 45 after 150 days. Increasing *β* increased the value of cumulative infected humans to 1,274 for *β* = 2.5. Therefore, the number of cumulative infected humans is very low compared to cumulative infected humans for similar values of *β* with *P*_0_ = 0.7. In addition, there is no pronounced single peak, while maximum simultaneously infected people goes to around 400 for the highest-used value of *β*. Therefore, decreasing *P*_0_ reduces the number of infected humans and, thus, the severity of EVD spreading.

**Figure 10:**
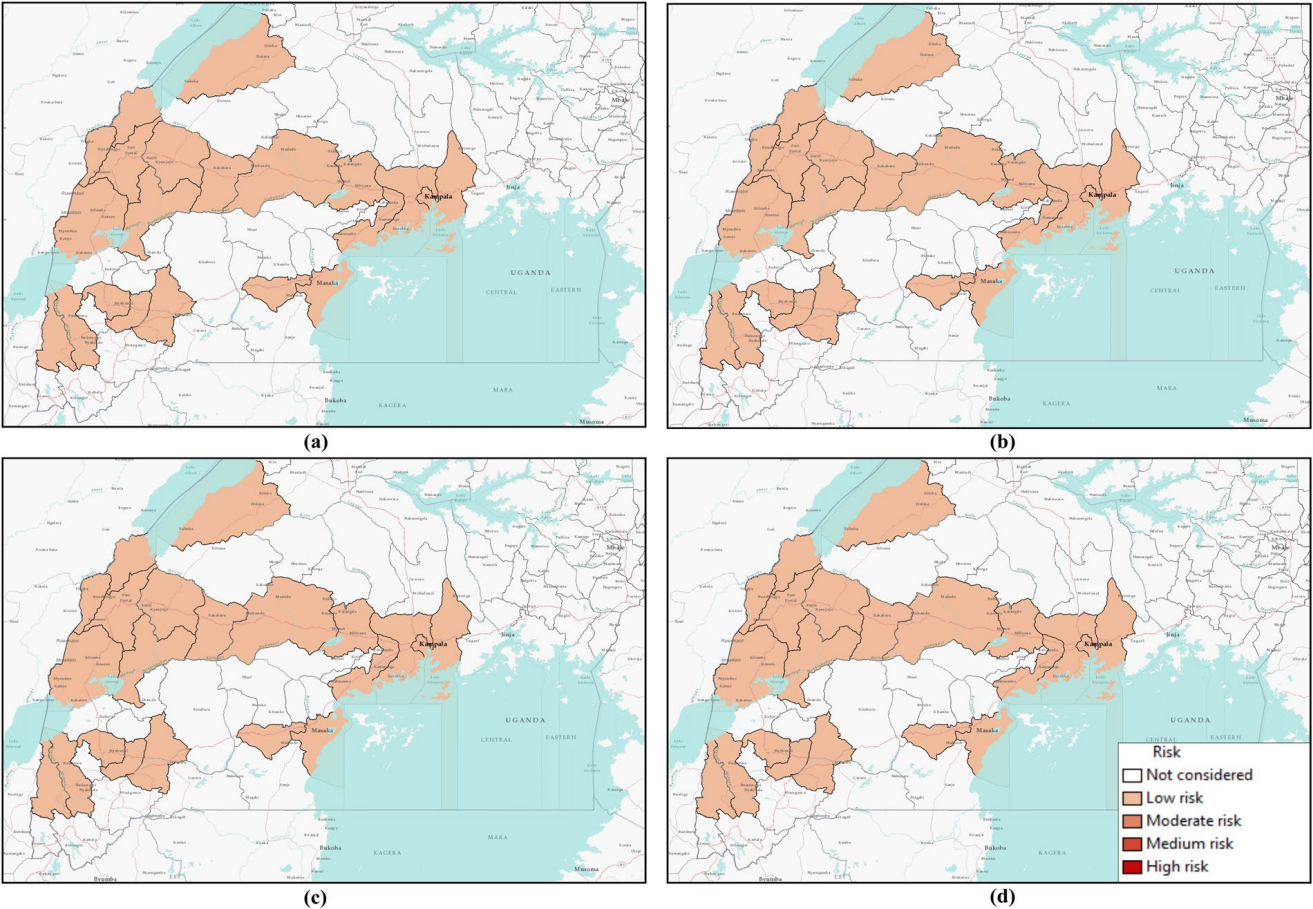
Risk map of Ebola spreading within selected 23 districts in Uganda for *P*_0_ = 0.1, *γ* = 0.5, and (**a**) *β* = 0.2, (**b**) *β* = 0.5, (**c**) *β* = 1.7, (**d**) *β* = 2.5. The map is colour coded according to the risk of Ebola spreading.

Figure 10 shows risk maps for *P*_0_ = 0.1 and *γ* = 0.5. It is evident from the maps that all districts in our network are at low risk for EVD spreading for selected values of *β*.

**Figure 11:**
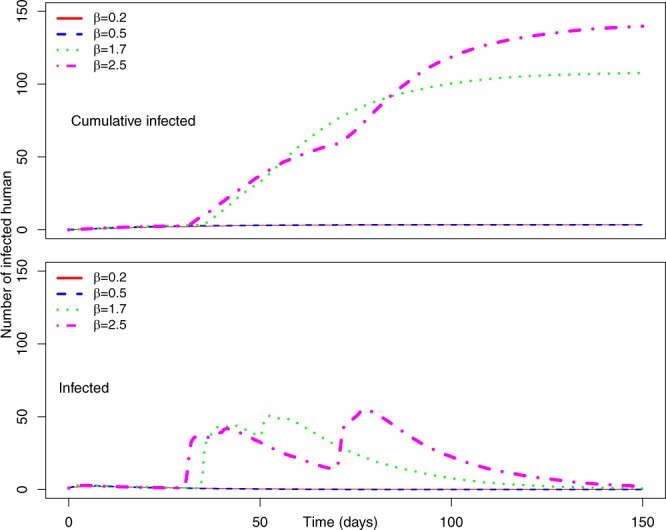
(Top) Average number of cumulative infected and (bottom) average number of infected humans in the Uganda Ebola network for *P*_0_ = 0.1, *γ* = 0.1 –four different colors represent the average number of infected humans for four values of *β*. Red, blue, green, and magenta lines correspond to the number of infected humans for *β* = 0.2, 0.5, 1.7, and 2.5, respectively.

Further decreasing the value of *γ* to 0.1 decreases the cumulative number of infected humans as well as peak infection size. Figure 11 shows that when *β* is 0.2 and 0.5, the EVD does not spread at all and stays within the outbreak location with only two to three infected persons. However, increasing the value of *β* increases the number of infected humans, but the cumulative number of infected remains less than 150 even for our highest value of transmission rate (*β* = 2.5).

**Figure 12:**
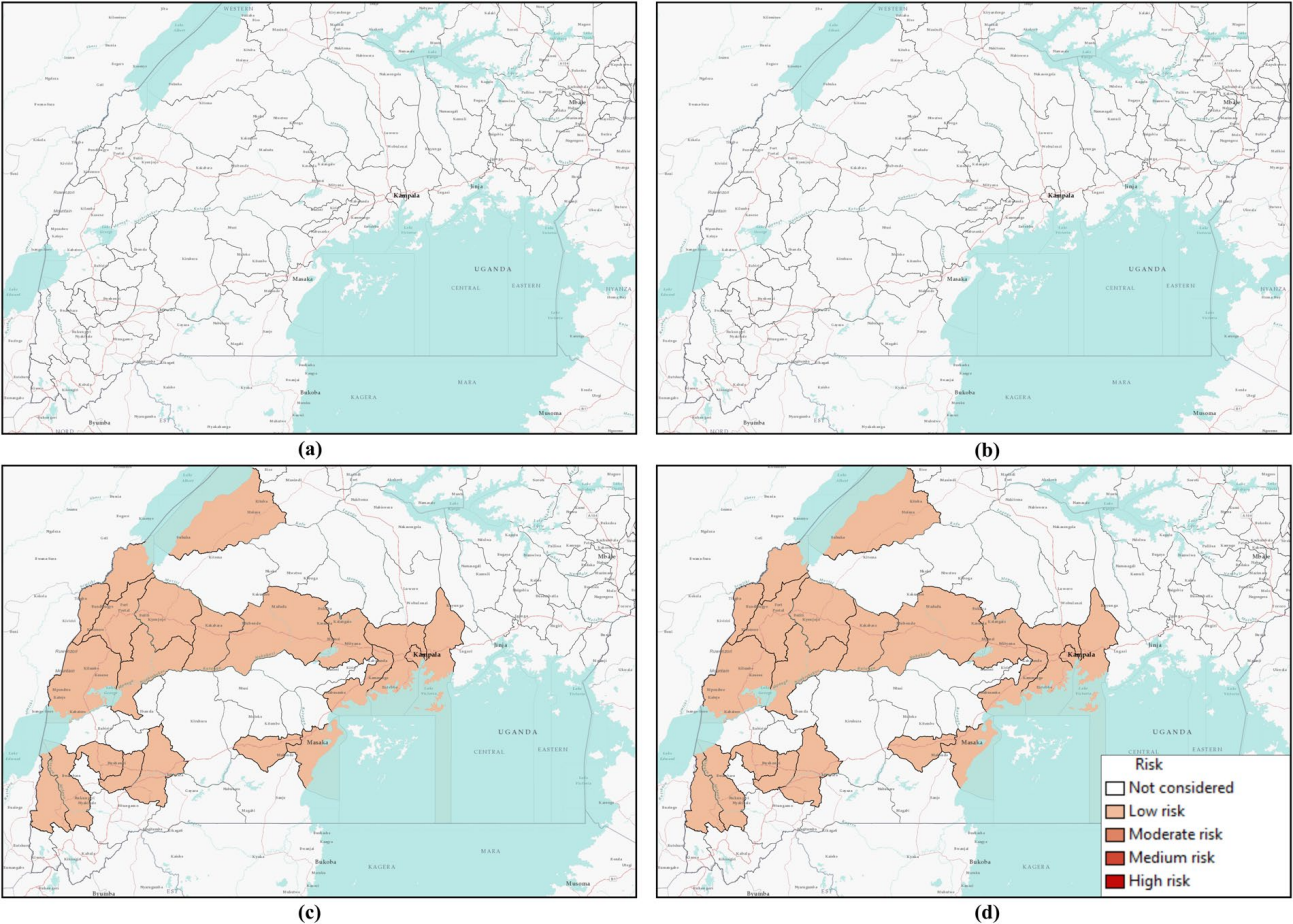
Risk map of Ebola spreading within selected 23 districts in Uganda for *P*_0_ = 0.7, *γ* = 0.5, and (**a**) *β* = 0.2, (**b**) *β* = 0.5, (**c**) *β* = 1.7, (**d**) *β* = 2.5. The map is colour coded according to the risk of Ebola spreading.

Figure 12 shows no risk of EVD spreading for *β* = 0.2 and 0.5. However, with increasing *β*, all our districts are at low risk of EVD spreading.

Summarizing simulation results and risk maps presented in Figs 6–9, we can see a significant reduction in the epidemic size and simultaneously infected humans when *P*_0_ = 0.1. Therefore, a reduction in probability of pathogen transfer via a potential link i.e. reduced physical contact between humans while they are active/mobile greatly reduce the number of infected as well as severity of EVD. It also reduces the risk of EVD spreading.

EVD spreads from physical contact with bodily fluids such as blood, feces, vomit, saliva, mucus, tears, breast milk, urine, semen, sweat, etc. from infected persons. Therefore, a susceptible person can only be infected with EVD if he or she comes in contact with these bodily fluids from an infected human. So, if infected people are identified and moved to quarantine, and are restricted from travel, then the infection can be contained. However, non-hospitalized people during early symptomatic stages keep moving for their daily lives and spread the infection to people they come in contact with. Early detection in countries where people are not very health conscious is challenging. Therefore, when an infected case is found, if human movement is reduced significantly, the spread can be contained. However, this is not practical as human movement cannot be controlled.

The activity rate (*γ*) can be translated to human movement within the network, therefore an increasing activity rate means increased but shorter duration of human movement. From all simulation results, it is evident that an increase in activity rate increases epidemic size as well as speed of the infection to reach its peak. Therefore, an increasing activity rate means severe and faster spreading EVD. Simulation results suggest that short duration but frequent human movement results in a greater number of infected humans and a higher risk of EVD spreading than longer duration but less frequent movement.

The probability at which infection spreads to susceptible people via a potential contact (*P*_0_) has obvious impacts on epidemic size. Increasing *P*_0_ increases epidemic size irrespective of the activity rate *γ*, which is evident from Figs 2, 4, 6 and 8. Comparing Figs 5 and 9, as well as Figs 7 and 11, for similar values of the activity rate, decreasing *P*_0_ decreases the number of infected humans significantly. *P*_0_ can be translated to the probability of physical contact of infected mobile humans with others. Therefore, our simulation results conform with another mitigation strategy against EVD spreading, which is to stay away from contact with people whose status with respect to EVD is not known. The lower value of *P*_0_ can be achieved by minimizing physical contact among people during movement/travel. If people are aware of the risk of EVD spreading in their area and keep themselves from physical contact with others, it will significantly reduce infected cases if there is an outbreak.

Risk maps shows some districts, as shown in Tables 3 and 4, are at higher risk of Ebola spreading in our specific network scenario. The risk assessment provides important information for Ebola preparedness, which includes setting up medical facilities as well as employing different preventive measures against disease spreading. However, resources are limited and it is always essential to find a way to utilize resources in a fruitful way. Yet our risk only showed examples of our developed risk assessment method where we have used very limited generalized movement data. Although the proposed risk assessment method has the capability to provide guidelines for public health people, it requires more accurate movement data to be used practically in the field. Future work will include an evaluation of the impact of major EVD intervention pillars (i. e., coordination, vaccination, surveillance, risk communication, case management, and safe burials) on risk assessment, to provide a realistic picture under multiple scenarios.

## Conclusions

We present a novel method for risk assessment based on a two-layer temporal network. This method has the ability to assess the risk of EVD spreading when accurate network data is available and can be an important tool for public health people during an outbreak. We demonstrate an application of our developed method using a two-layer temporal network formulated with generic and incomplete movement data in Uganda. Simulation results from this two-layer temporal network confirm that reduced physical contact with people while travelling, as well as taking other preventive measures, decreases the risk of EVD spreading. Simulations also show some districts are at higher risk than others in the scenario considered. This provides public health personnel direction for prioritizing their efforts to limit EVD spreading during an outbreak. However, assessed risks are crucially dependent on the network structure and can only be fully trusted for resource allocation once accurate, individual-level movement data in time and space are available.

## Supplementary information

Supporting information

Supporting code and data

## Data Availability

The authors confirm that the data and the core code supporting the findings/analysis of this study are available within the article and its Supplementary Materials.
