# Supplementary material for: Risk assessment of Ebola virus disease spreading in Uganda using a two-layer temporal network
Source: Sci Rep. 2019 Nov 5;9:16060. doi: 10.1038/s41598-019-52501-1 (PMC6831630; doi:10.1038/s41598-019-52501-1)
Supplement: Supplementary file 1 — Supporting information [file 41598_2019_52501_MOESM1_ESM.docx]

Supplementary Information

Risk assessment of Ebola virus disease spreading in Uganda using a two-layer temporal network

Mahbubul H Riad, Musa Sekamatte, Felix Ocom,

Issa Makumbi, and Caterina M Scoglio


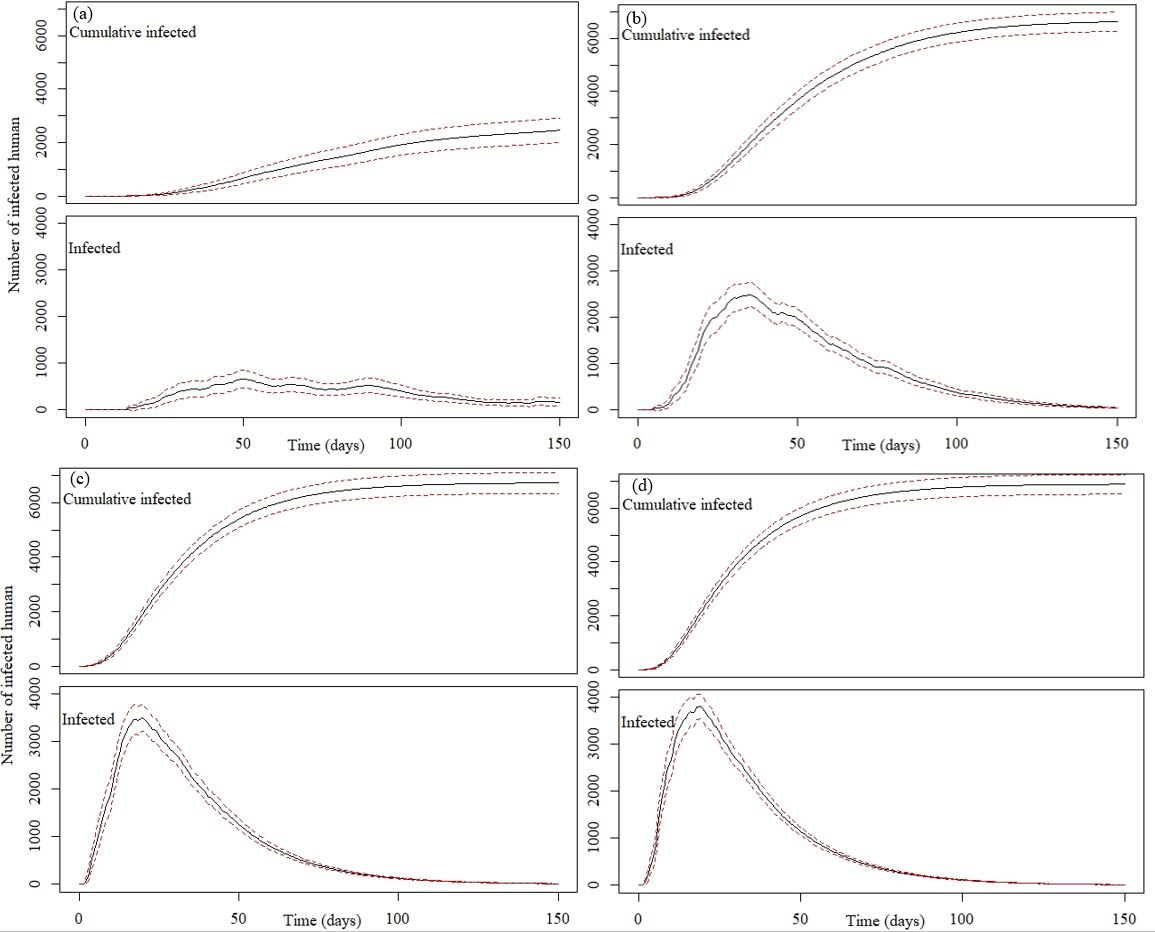


**Supplementary Figure 1:** Number of infected individuals in the Uganda Ebola network with 95% confidence interval and *P_0_*=0.7, *γ*=0.5, and for (a) *β*=0.2, (b) *β* =0.5, (c) *β* =1.7, and (d) *β* = 2.5.


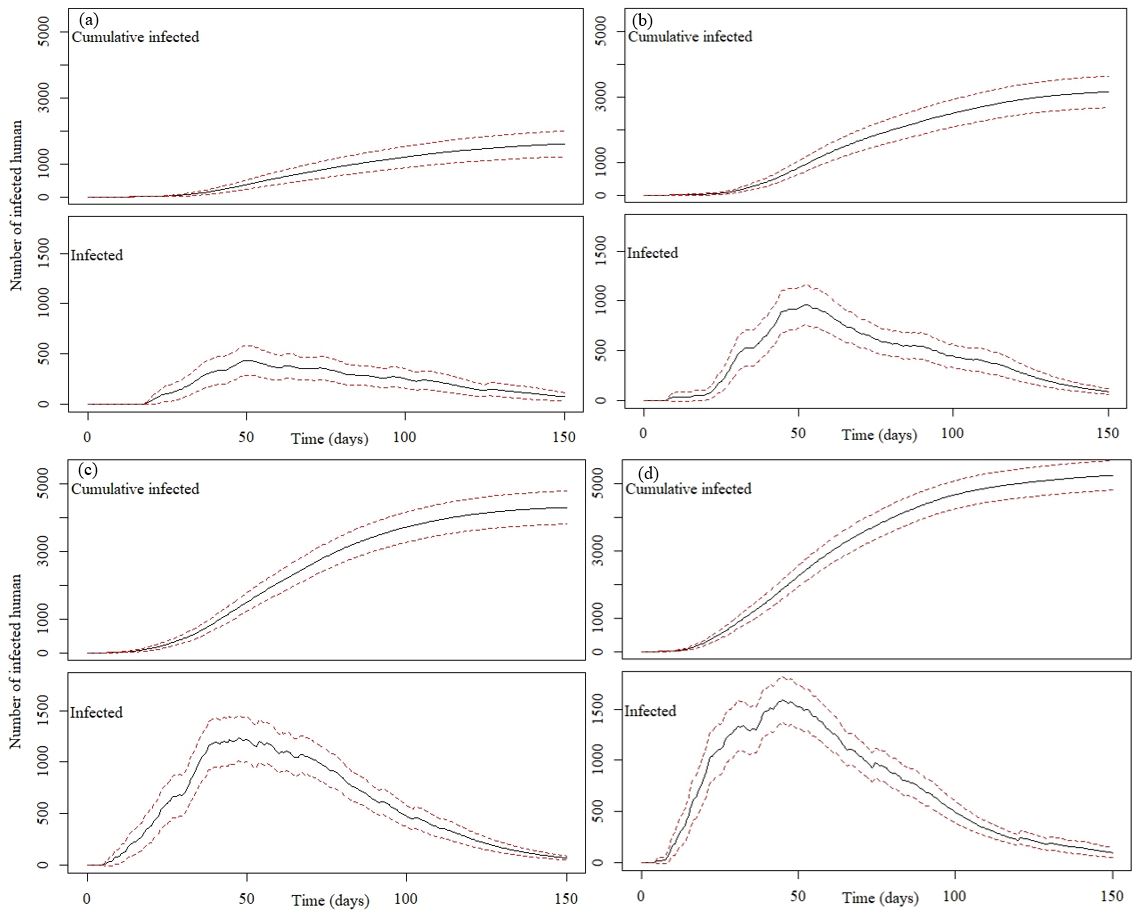


**Supplementary Figure 2:** Number of infected individuals in the Uganda Ebola network with 95% confidence interval and *P_0_*=0.7, *γ*=0.1, and for (a) *β*=0.2, (b) *β* =0.5, (c) *β* =1.7, and (d) *β* = 2.5.


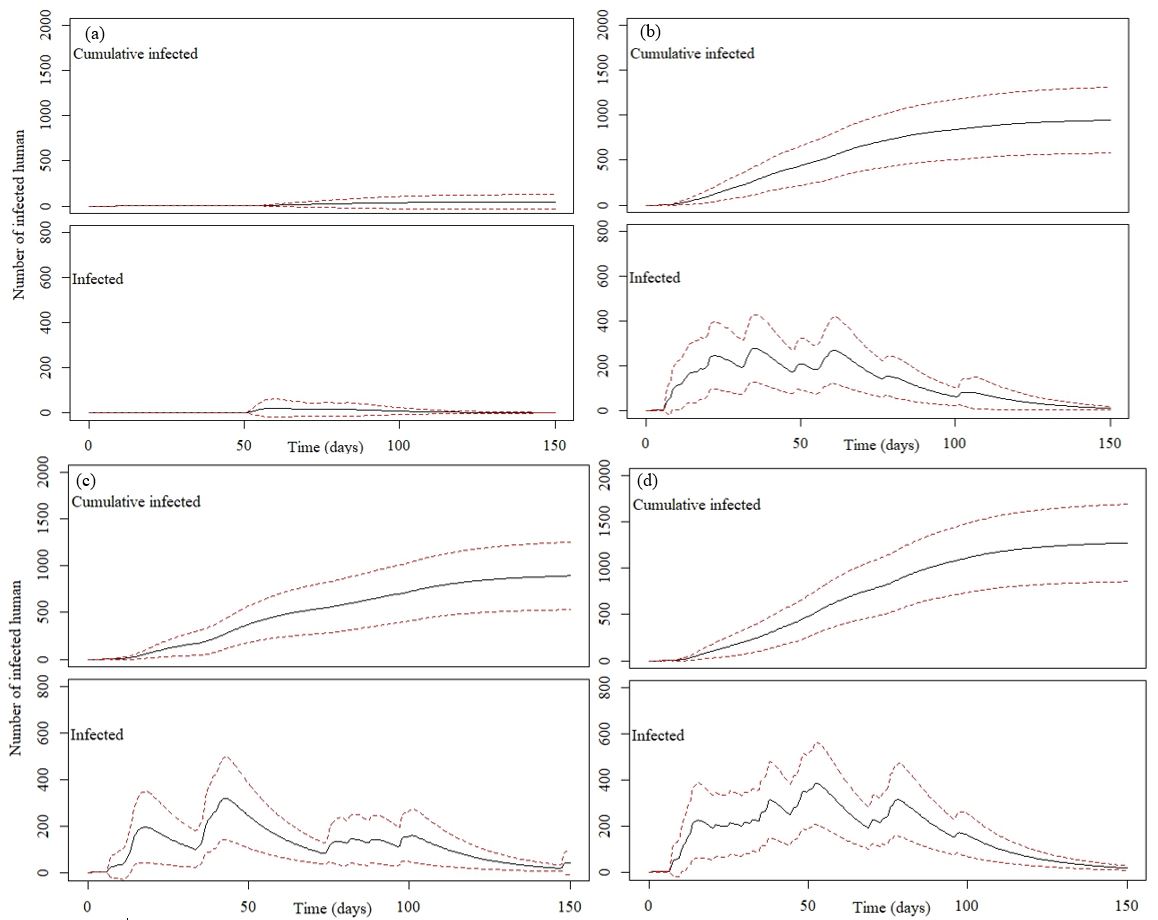


**Supplementary Figure 3:** Number of infected individuals in the Uganda Ebola network with 95% confidence interval and *P_0_*=0.1, *γ*=0.5, and for (a) *β*=0.2, (b) *β* =0.5, (c) *β* =1.7, and (d) *β* = 2.5.


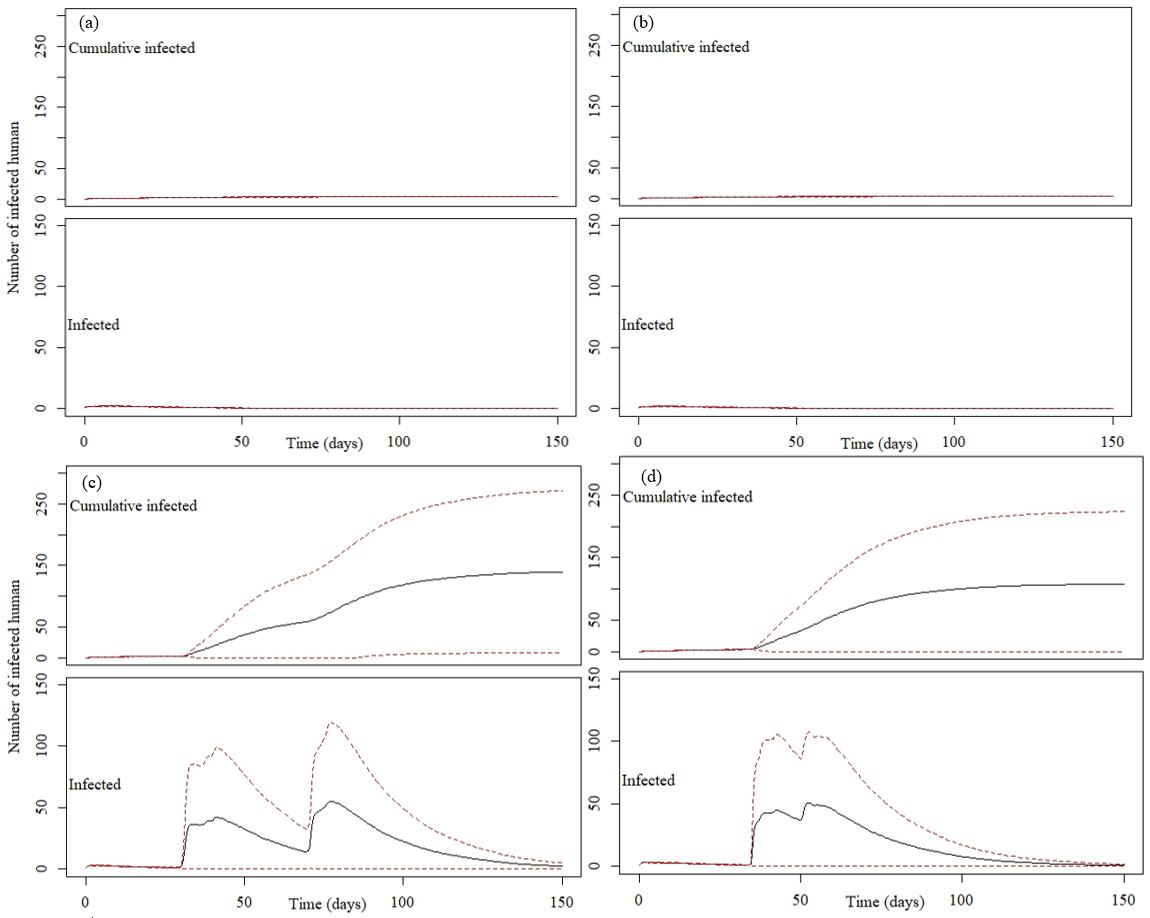


**Supplementary Figure 4:** Number of infected individuals in the Uganda Ebola network with 95% confidence interval and *P_0_*=0.1, *γ*=0.1, and for (a) *β*=0.2, (b) *β* =0.5, (c) *β* =1.7, and (d) *β* = 2.5.

**
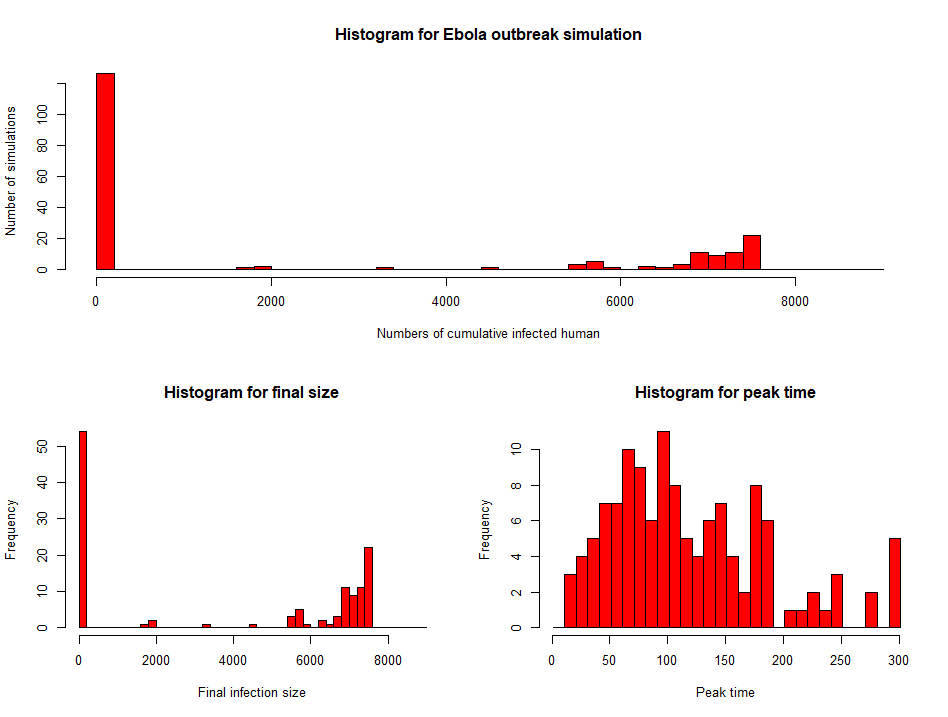
**

**Supplementary Figure 5:** Histogram for number of simulations with the cumulative infected human, final size of the outbreak and peak infection time with *β* = 0.2, *γ* =0.5 and *P_0_* = 0.7.

**
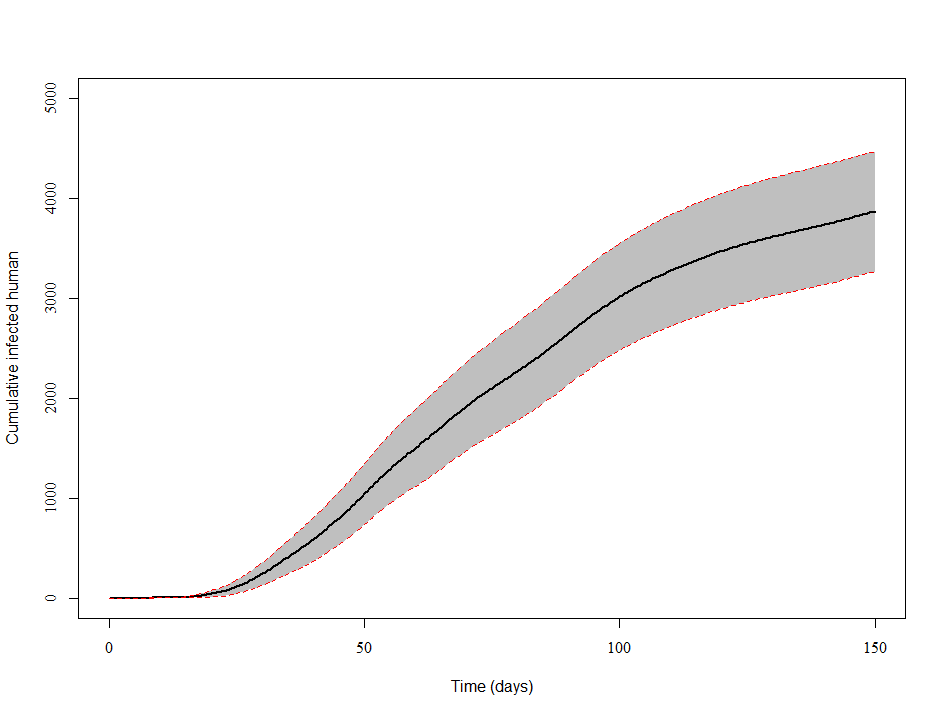
**

**Supplementary Figure 6:** Numbers of cumulative infected human with time for simulations who resulted in an outbreak and *β* = 0.2, *γ* =0.5 and *P_0_* = 0.7.

**
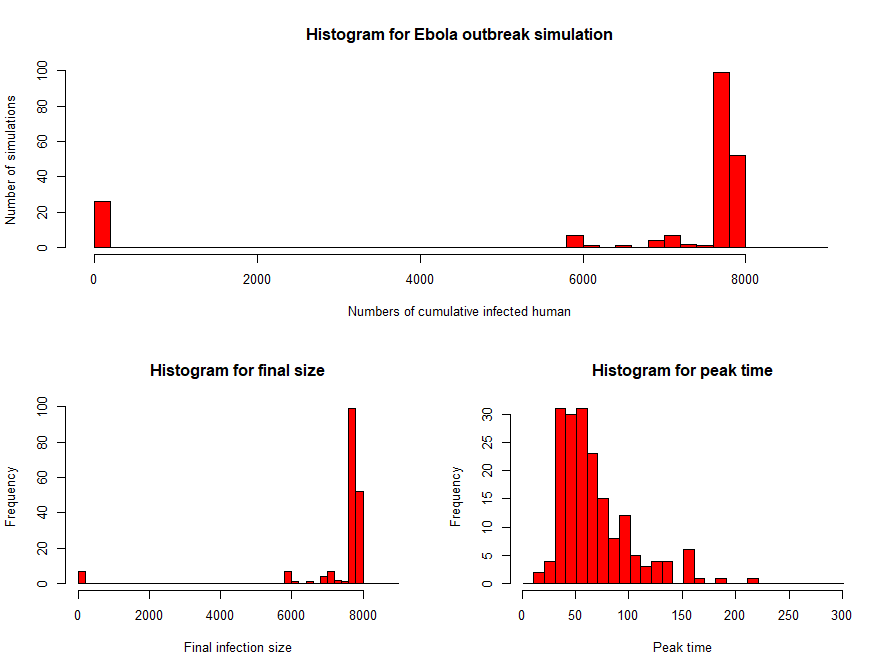
**

**Supplementary Figure 7:** Histogram for number of simulations with the cumulative infected human, final size of the outbreak and peak infection time with *β* = 0.5, *γ* =0.5 and *P_0_* = 0.7.

**
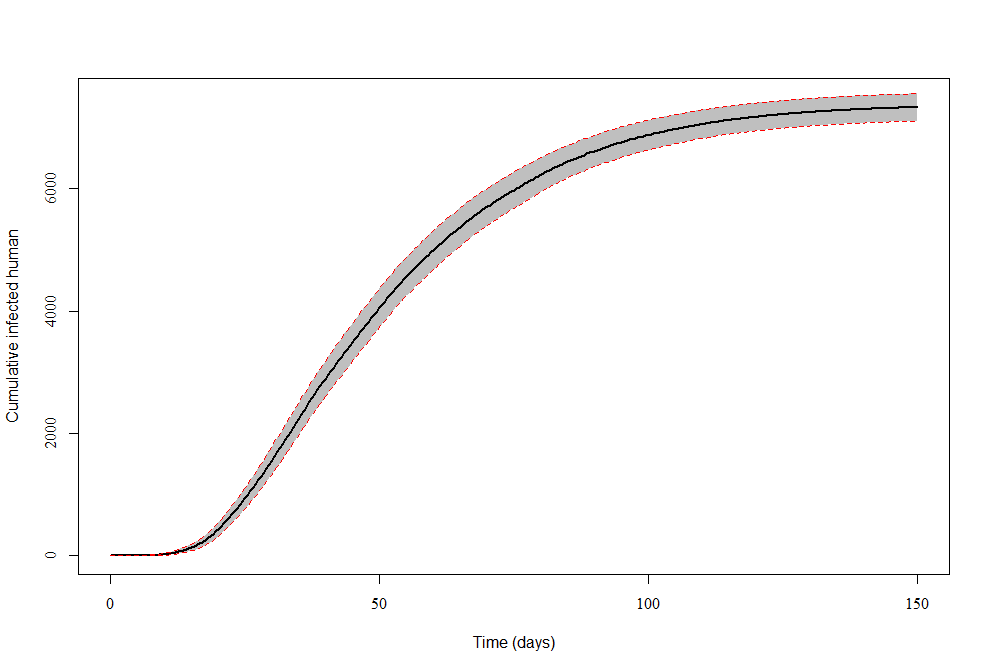
**

**Supplementary Figure 8:** Numbers of cumulative infected human with time for simulations who resulted in an outbreak and *β* = 0.5, *γ* =0.5 and *P_0_* = 0.7.

**
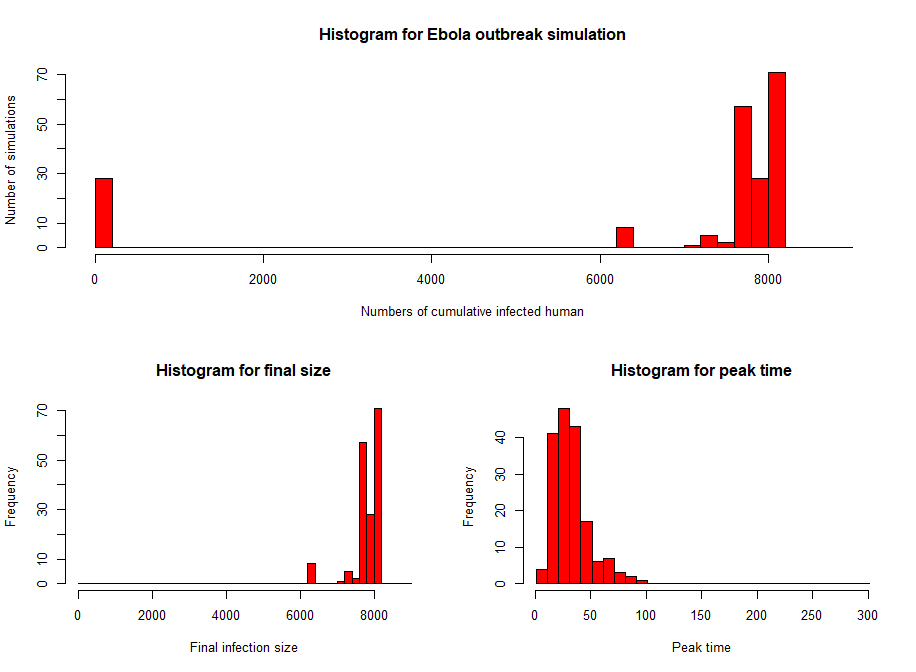
**

**Supplementary Figure 9:** Histogram for number of simulations with the cumulative infected human, final size of the outbreak and peak infection time with *β* = 1.7, *γ* =0.5 and *P_0_* = 0.7.

**
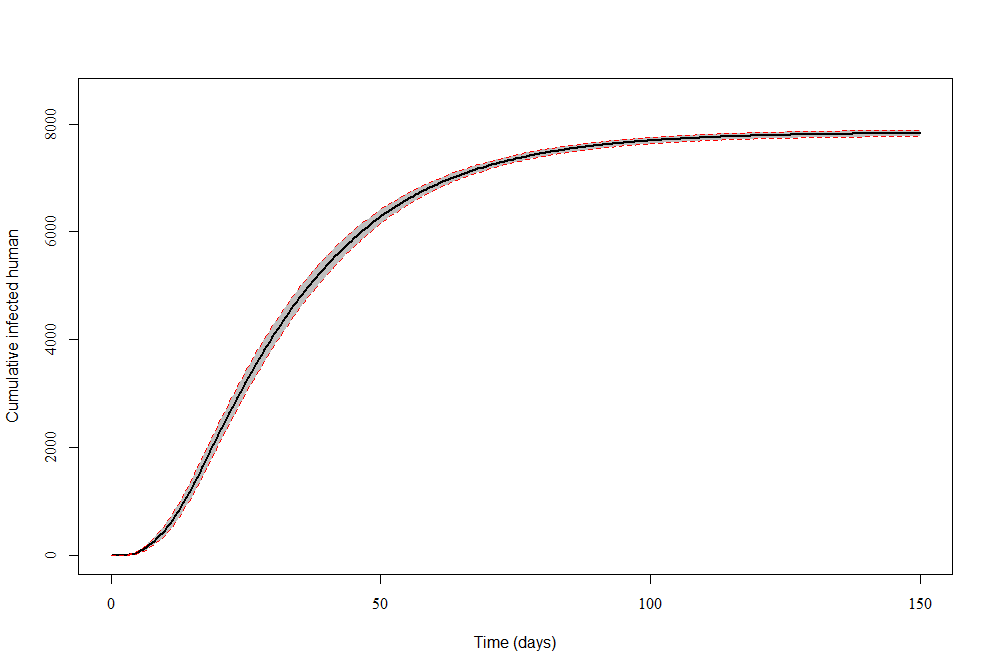
**

**Supplementary Figure 10:** Numbers of cumulative infected human with time for simulations who resulted in an outbreak and *β* = 1.7, *γ* =0.5 and *P_0_* = 0.7.

**
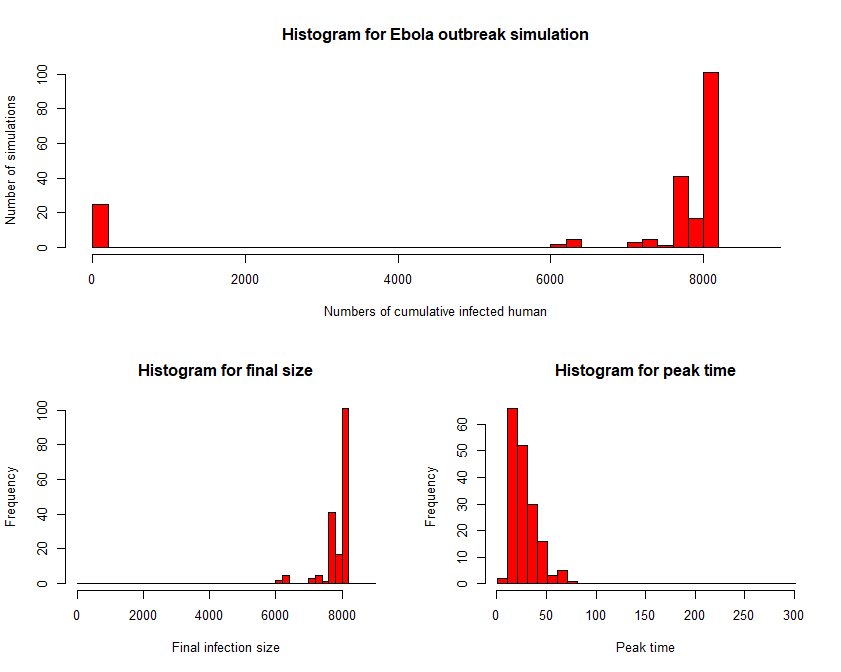
 Supplementary Figure 11:** Histogram for number of simulations with the cumulative infected human, final size of the outbreak and peak infection time with *β* = 2.5, *γ* =0.5 and *P_0_* = 0.7.

**
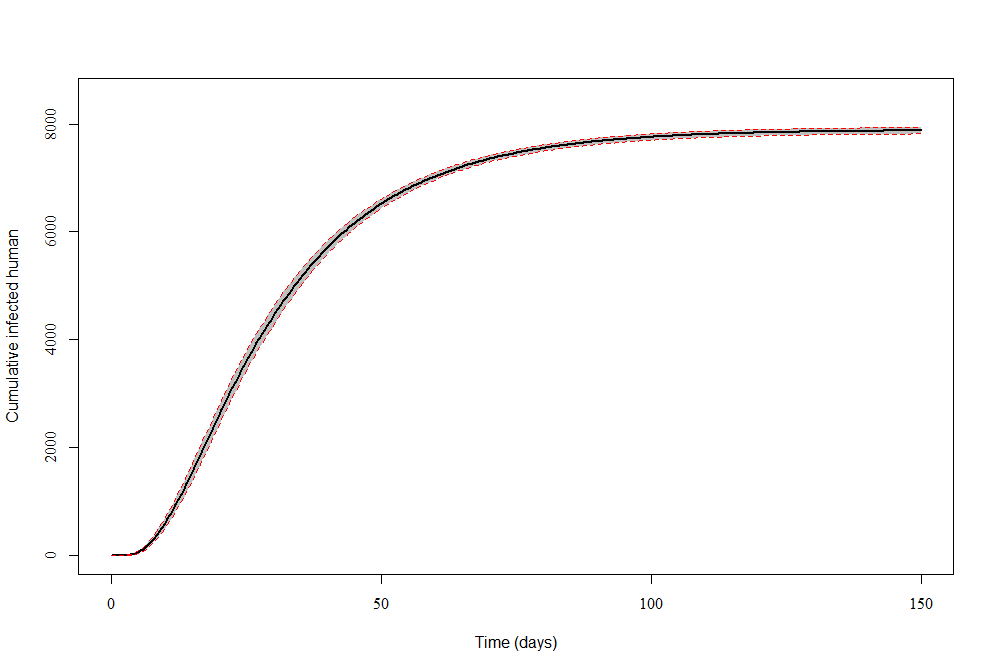
**

**Supplementary Figure 12:** Numbers of cumulative infected human with time for simulations who resulted in an outbreak and *β* = 2.5, *γ* =0.5 and *P_0_* = 0.7.

**Supplementary Table 1:** Confidence interval of final size for different parameter sets

| *P_0_* | *γ* | *β* | Average final size | 95% Confidence interval | |
| --- | --- | --- | --- | --- | --- |
|  |  |  |  | Lower | Upper |
| 0.7 | 0.5 | 0.2 | 2459.39 | 2001.625 | 2917.155 |
|  |  | 0.5 | 6634.755 | 6274.522 | 6994.988 |
|  |  | 1.7 | 6731.755 | 6351.206 | 7112.304 |
|  |  | 2.5 | 6891.805 | 6526.327 | 7257.283 |
| 0.7 | 0.1 | 0.2 | 1607.65 | 1217.644 | 1997.656 |
|  |  | 0.5 | 3157.855 | 2675.505 | 3640.205 |
|  |  | 1.7 | 4303.03 | 3812.693 | 4793.367 |
|  |  | 2.5 | 5249.93 | 4810.483 | 5689.377 |
| 0.1 | 0.5 | 0.2 | 3.47 | 1.3 | 128.227 |
|  |  | 0.5 | 944.9125 | 578.8293 | 1310.996 |
|  |  | 1.7 | 944.9125 | 578.8293 | 1310.996 |
|  |  | 2.5 | 1274.794 | 856.3116 | 1693.276 |
| 0.1 | 0.1 | 0.2 | 3.43 | 3.13 | 3.80 |
|  |  | 0.5 | 3.47 | 3.1895 | 3.60 |
|  |  | 1.7 | 107.63 | 6.8057 | 223.691 |
|  |  | 2.5 | 139.73 | 7.6804 | 271.77 |
