# Supplementary material for: Risk assessment of Ebola virus disease spreading in Uganda using a two-layer temporal network
Source: Sci Rep. 2019 Nov 5;9:16060. doi: 10.1038/s41598-019-52501-1 (PMC6831630; doi:10.1038/s41598-019-52501-1)

Page 1 - Overview (Results from Kasese FGDs, Uganda)

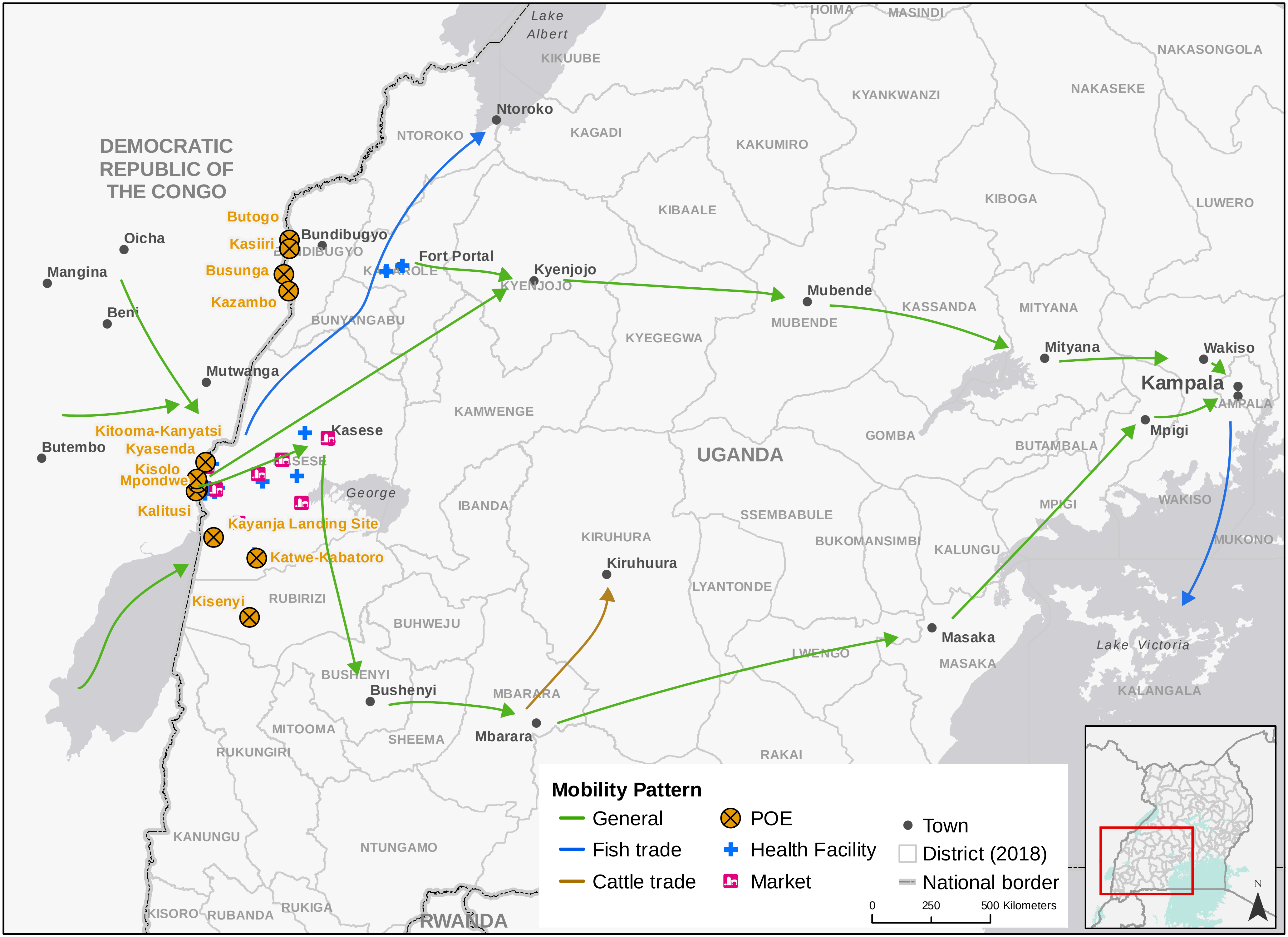

Page 2 - Kasese Area Detail (Results from Kasese FGDs, Uganda)

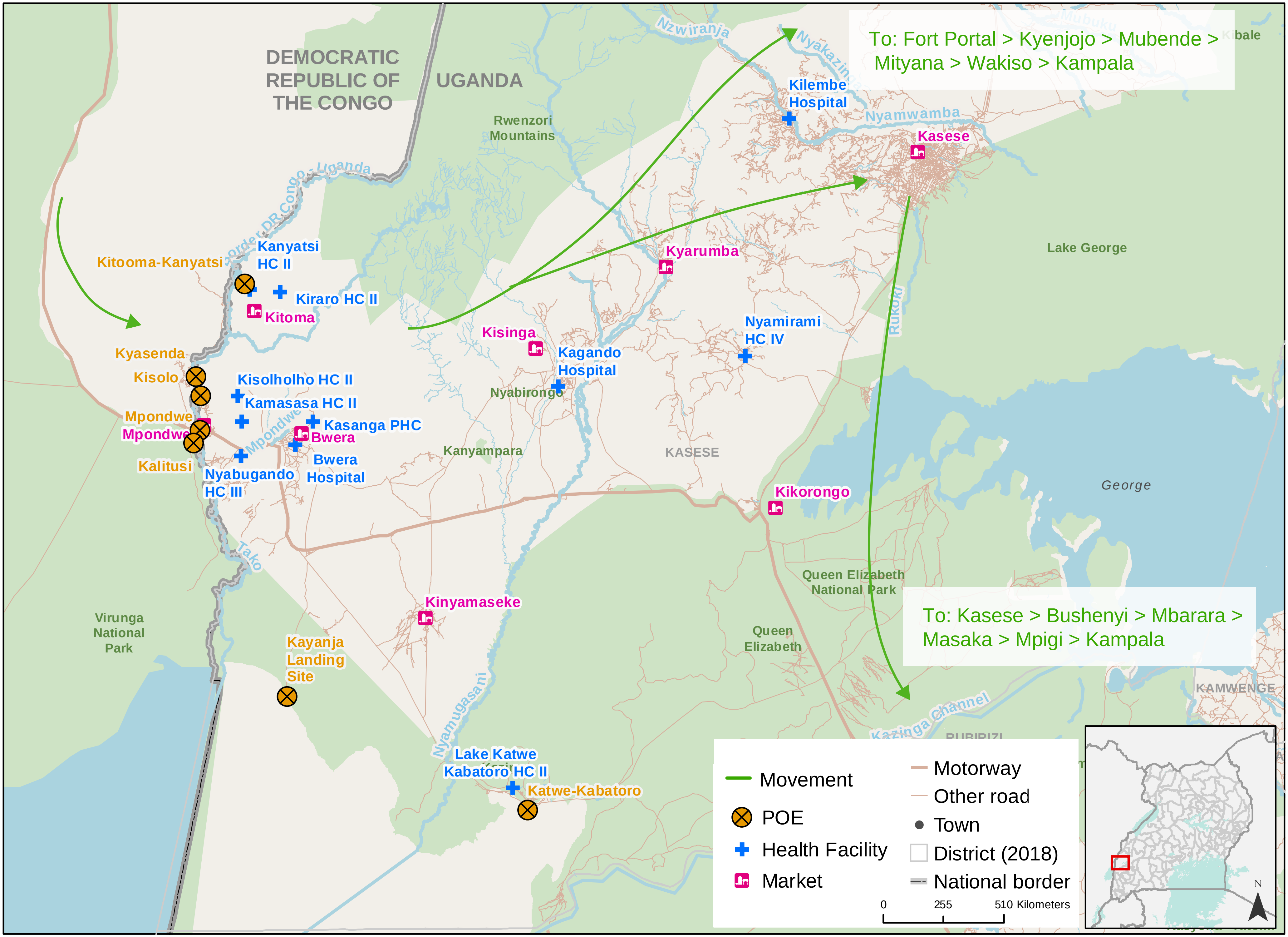

Page 3 - Kampala Area Detail (Results from Kasese FGDs, Uganda)

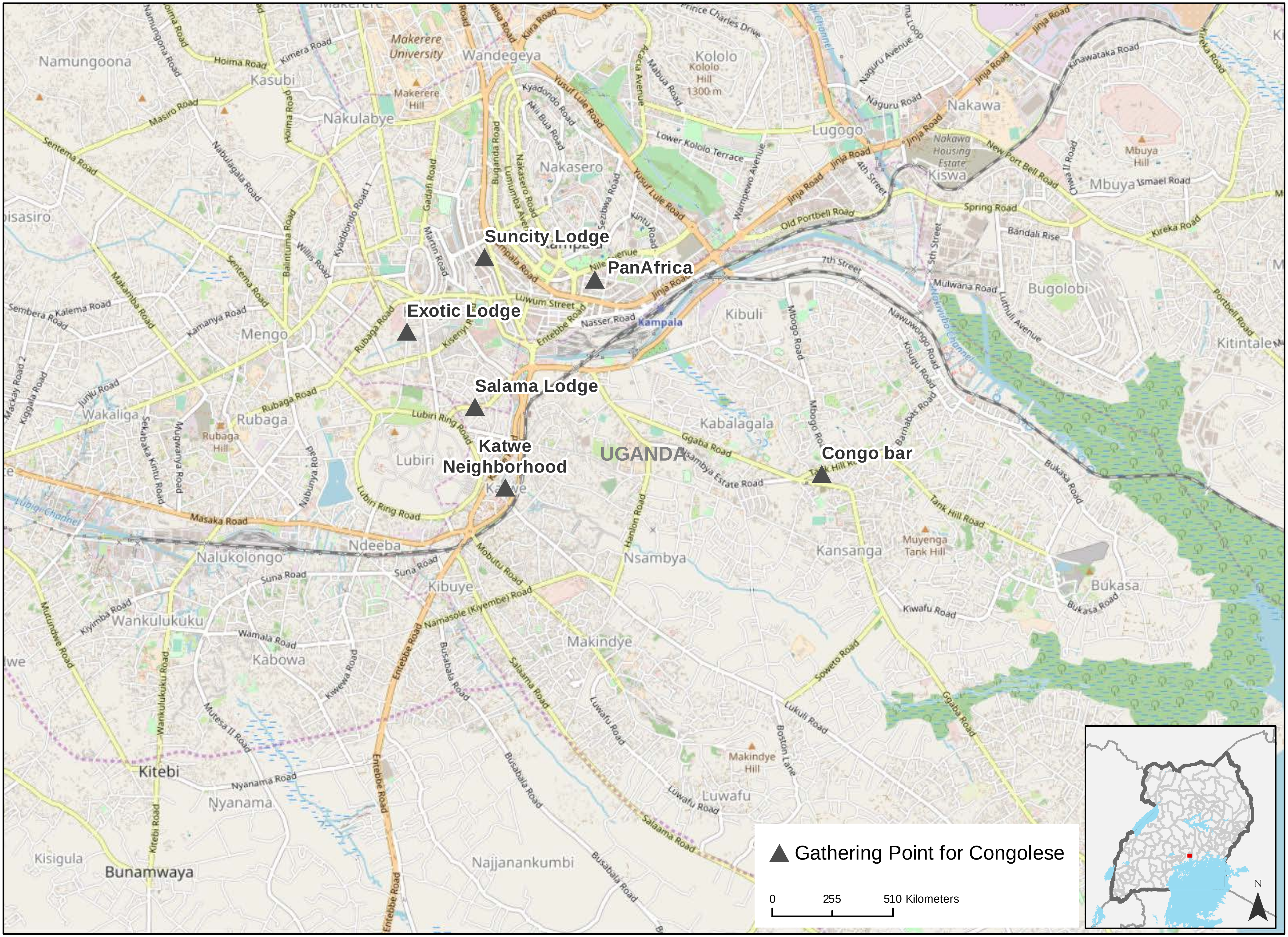

Page 4 - Bundibugyo Area Detail (Results from Bundibugyo FGDs, Uganda)

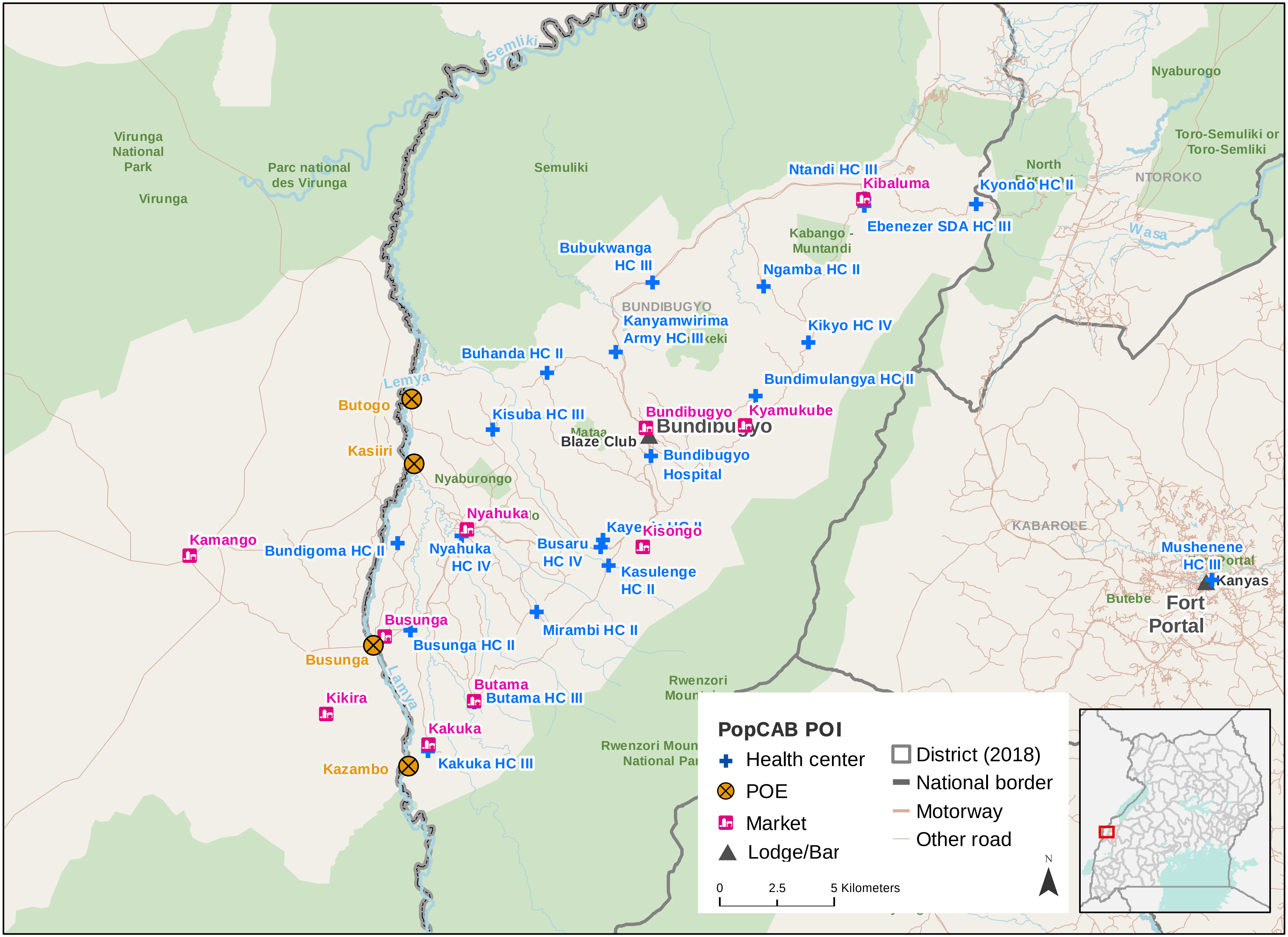

Page 5 - Kabarole Area Detail (Results from Kabarole FGDs, Uganda)

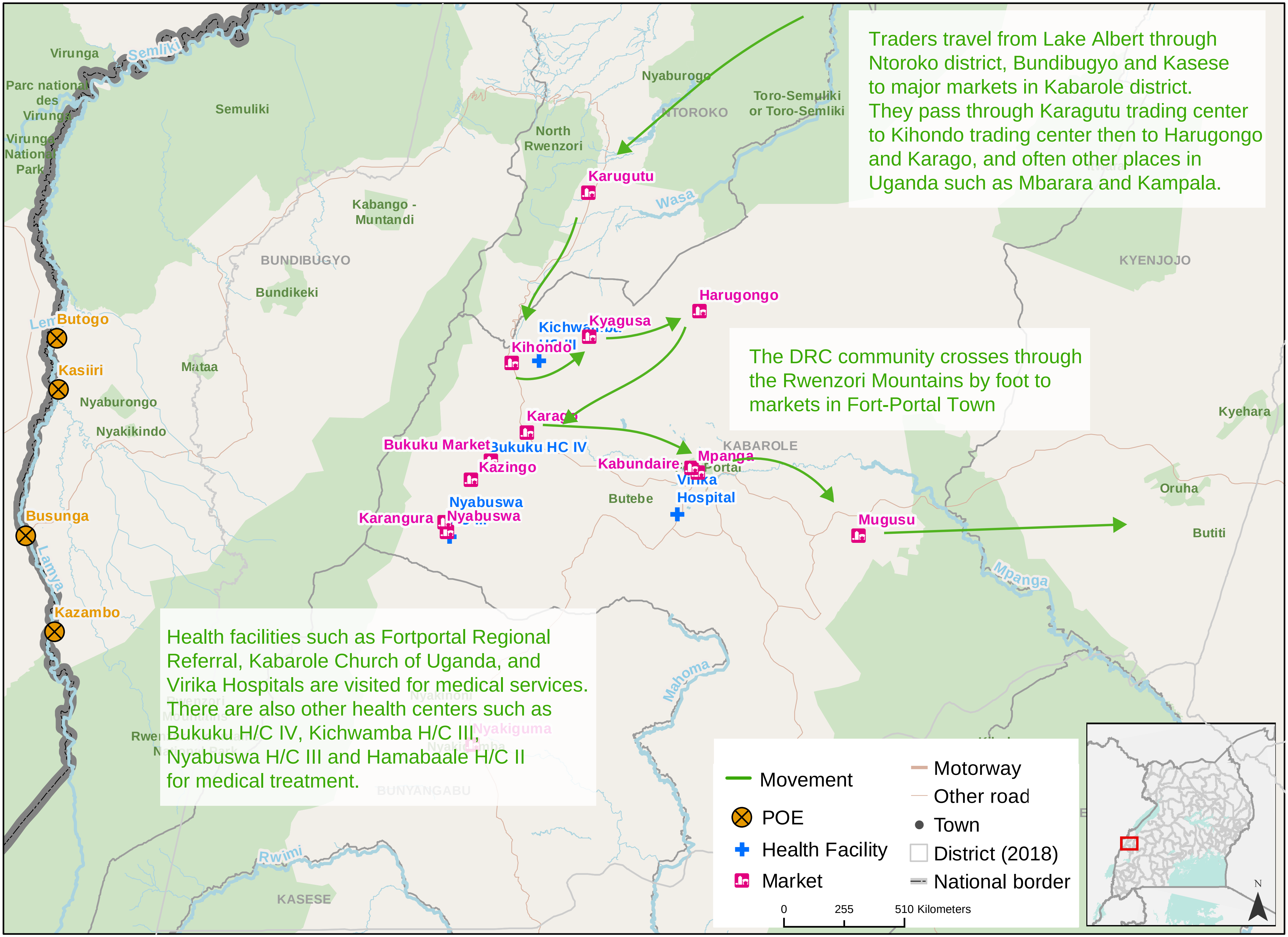

Supplement: Supplementary file 2 — Supporting code and data [file 41598_2019_52501_MOESM2_ESM.zip › Supporting code data/Map used for network creation.pdf]
