# Supplementary material for: Risk assessment of Ebola virus disease spreading in Uganda using a two-layer temporal network
Source: Sci Rep. 2019 Nov 5;9:16060. doi: 10.1038/s41598-019-52501-1 (PMC6831630; doi:10.1038/s41598-019-52501-1)

List of Functions

- **GEMF_SIM:** This function generate a realization of epidemic process over a network.
- **Post_Population:** Analyzes the output of *GEMF_SIM* function and calculates the population of each comportment(nodestate) through time.
- **Post_NodesState:** After executing *GEMF -SIM*, *Post_NodesState* function can be used in order to obtain the state of each individual node of network at different time instants.
- **GEMF_SIM_Prob:** This function generates several realizations of the epidemic process. The output of *GEMF_SIM_Prob* can be used to estimate the probability distribution of finding a node in different nodestates as a function of time.
- **Net_Import:** Reads a txt file that contains the information for a single layer network and produces the parameter *Net* which can be fed into the *GEMF_SIM* function as a input argument.
- **NetCmbn:** This function combines single-layer networks into one multilayer network.
- **Para_SIS:** Generates an argument *Para* from the specification of the SIS epidemic model. *GEMF_SIM* function can use the output of *Para_SIS* as an input argument.
- **Para_SAIS_2layer:** Generates the argument *Para* from the specification of the SAIS epidemic model over a two-layer network. *GEMF_SIM* function can use the output of *Para_SIS_2layer* as an input argument.
- **NetGen_Geo:** Generates *Net* parameter for the random geometric graph.
- **NetGen_ER:** Generates *Net* parameter for the Erdos-Renyi random graph.

Description

- ## GEMF_SIM

  lst=GEMF_SIM(Para,Net,x0,maxNumevent,Runtime,N)
  GEMF_SIM function uses the algorithm described in [cite] in order to generate one realization of a epidemic process over a network. In general each node of network can be in one of the M states (compartments) and the transition to another state depends on the state of the node and the state of its neighbors in different layers.

  Input Arguments

  1. **Para** The input *Para* is a list that contains the parameters which define the spreading model, *Para=list(M,q,l,A_d,A_b)*. Here M and l are the number of compartments and the layers, respectively. q is a $1 \times l$ matrix where $q[1,s]$ is the influencer compartment for layer s. The nodal based transition matrix ,*A_d*, is a $M \times M$ matrix with elements $A\_d[i,j]$ that specifies the the nodal based transition rate from compartment i to j. The other element of *Para* is *A_b* which is a $M \times M \times l$ array. The element $A\_b[i,j,s]$ is the transition rate of a node from compartment i to j if it has a neighbor in layer s and the state of the neighbor is $q[1,s]$.
  2. **Net** Here *Net* is a list of elements that specifies the contact network, *Net=list(Neigh,I1,I2)*. I1 and I2 are $l \times N$ matrices where l is the number of layers and N is the number of nodes in the network. *Neigh* is a list that contains l matrices where each matrix has 2 rows. The neighbors of node n in the layer s are the element of the vector $v = Neigh[[s]][1, I1[s,n] : I2[s,n]]$.These are the nodes that can be potentially affected by the node n. Moreover the weight of the link between node n and its neighbors, obtained from vector v, are $Neigh[[s]][2, I1[p,n] : I2[p,n]]$ respectively.
  3. **maxNumevent**
  4. **Runtime**
     maxNumevent and Runtime determine how the simulation terminates. In fact simulation will stop if the number of event reaches *maxNumevent* or the total evolution time reaches the *Runtime*
  5. **N** is the number of node in the network

6. **x0** is a vector with the length N that stores the initial state of each node. For example if the node n is initially in compartment m then $x0[n] = m$. *GEMF_SIM* uses $x0$ as a initial condition for the network.

Output Argument The output of *GEMF_SIM* is a list which contains the summary of simulation. *lst=list(ts,n_index,i_index,j_index,Tf,lasteventnumber)*

1. **lasteventnumber**
2. **Tf**
   The simulation stops if any of these cases happen: (a) the number of events reaches the input argument *maxNumevent.* (b) the total evolution time reaches the input argument *Runtime.* (c) the system reaches equilibrium, in a sense that the rate of changes in the system is smaller than a specified threshold. Hence it is possible that the total number of events would be smaller than *maxNumevent* or the time span of simulation would not become *Runtime.* In general, the output arguments *lasteventnumber* and *Tf* are the total total number of events and the time duration of simulation.
3. **ts** is a vector with a length which is equal to the input parameter *maxNmevent* and stores the time interval between the events[cite paper]. For example, $ts[i]$ is the time interval between $(i-1)th$ and $(i)th$ events. Since the number of events that happen in the simulation equals the output argument *lasteventnumber*, all the elements of *ts* with index i greater than *lasteventnumber* are zero. $ts[i] = 0 \; if \; i > lasteventnumber$. Moreover $Tf = \sum_{i=1}^{lasteventnumber} ts[i]$.
4. **n_index**
5. **i_index**
6. **j_index**
   Considering the event based algorithm [cite paper]adopted for the simulation, we use the the output arguments *n_index, i_index* and *j_index* in order to keep the record of the events. These output arguments are vectors with the length equals to *maxNumevent.* The $(i)th$ event is a transition where the node $n\_index[i]$ changes its state from the compartment $i\_index[i]$ to the compartment $j\_index[i]$.

- ## Post_Population

lst2=Post_Population(x0,M,N,ts,i_index,j_index,lasteventnumber)
For one realization of the spreading process, generated by *GEMF_SIM* function, we can use the *Post_Population* function to obtain the number of the nodes, at different time instants, that have a specific state.

Input Arguments

1. **ts**
2. **i_index**
3. **j_index**
4. **lasteventnumber**
   The parameters *ts*, *i_index*, *j_index* and *lasteventnumber* are the output of *GEMF_SIM* function.
5. **x0** is a vector that stores the initial states of the nodes. This parameter is the same input argument used for *GEMF_SIM* function.
6. **M** is the number of node states.
7. **N** is the number of nodes in the network.

Output Argument The output of *Post_Population* function is a list that contains the numeric vector T and the *StateCount* matrix. *lst2=list(T,StateCount).*

1. **T** stores the times in the simulation that an event happened and it is calculated using the cumulative sum of *ts* as *T=c(0,cumsum(ts[1:lasteventnumber])).*
2. **StateCount** matrix has the dimension $M \times t$, where M is the number of node states and t is the length of the output vector *T*. The *StateCount* matrix stores the population of each comportment at the time-points specified by the elements of T. Thus, considering one realization of the spreading process using *GEMF_SIM*, the *StateCount[i,j]* is the number of nodes that were in the compartment i in the time interval $[T[j], T[j+1])$.

- **Post_NodesState**

lst3=Post_NodesState(x0,M,N,ts,n_index,j_index,lasteventnumber,timstp,Runtime)
For one realization of the spreading process, generated by *GEMF_SIM* function, Post NodesState function can be used to retrieve the state of each individual node at different time instants.

Input Arguments

1. **ts**
2. **n_index**
3. **j_index**
4. **lasteventnumber**
The parameters *ts*, *n_index*, *j_index* and *lasteventnumber* are the output of *GEMF_SIM* function.
5. **x0** is a vector that stores the initial states of the nodes. This parameter is the same input argument used for *GEMF_SIM* function.
6. **M** is the number of node states.
7. **N** is the number of nodes in the network.
8. **Runtime** is the same input parameter used for *GEMF_SIM* function.
9. **timstp** The *Post_NodesState* function uses the input parameter *timstp* to produce a sequence of time-points, Tr, from *t=0* to *t=Runtime* with the step-size equals to *timstp.* The function task is to retrieve the state of each node at the time instants specified by the elements of Tr.

Output Argument The output of *Post_NodesState* function is a list that contains the numeric vector *Tr* and the *nodstt* matrix. *lst2=list(Tr,nodStt).*

1. **Tr** is a sequence of time-points, from *t=0* to *t=Runtime* with the step-size equals to the input parameter *timstp.* The *Post_NodesState* function retrieves the states of each node at the time instants specified by the elements of Tr.
2. **nodStt** is a matrix that has $t \times N$ dimensions. Here t is the length of the output argument *Tr* and N is the number of nodes. *Nodstt[i,j]* is the state of node j at the time point *Tr[i].* Considering the algorithm used by *GEMF_SIM* function for the realization of the spreading process[cite paper], every time an event happens one of the nodes in the network changes its state. Assuming *T[j]* is the time when the *jth* event happens, the node states in the time interval *[T[j], T[j + 1])* does not change[1]. Hence the states of nodes at $Tr[i]$ is the same as the states of nodes in the time interval $[T[j], T[j+1])$ as long as $Tr[i]$ rests in the interval. If some of the time points in the sequence Tr are greater than the time, *Tf* [2], that last event happens, the state of nodes will be assigned as the same states that they had at *Tf.*

- **GEMF_SIM_Prob**

lst4=GEMF_SIM_Prob(Para,Net,X0,maxNumevent,Runtime,N,numrun,timstp,comp, drawfromprobdis,P0)
Using this function we can perform the Monte Carlo simulation of the stochastic spreading models over the network in order to obtain an estimation for the probability that a node occupies a specific compartment at different time instants. This function generates several realizations of the epidemic process and counts the occasions that a node occupies a compartment at a certain time point.

Input Arguments

1. **Para**
2. **Net**
3. **X0**
4. **maxNumevent**
5. **Runtime**

[1] T is the output argument of the *Post_Population* function
[2] *Tf* is the output of *GEMF_SIM* function

6. **N**
The parameters *Para,Net,X0,maxNumevent,Runtime,N* are the same input parameters defined for the *GEMF_SIM* function.
7. **numrun** is the number of the process's realizations that will be generated.
8. **timstp** is a numeric value that will be used as the step-size to produce a sequence of time-points, Tp, from *t=0* to *t=Runtime*. The function counts the occasions that a node occupies a compartment at a certain time point specified by the elements of Tp.
9. **comp** is a numeric vector with the node states as its elements. For example if we want the function count the number of times that a node assumes node states i and j ,*comp* should be a vector with i and j as its elements, $comp = c(i, j)$.
10. **drawfromprobdis** is a Boolean value. If we set the input parameter *drawfromprobdis=TRUE*, the function starts each realization of the stochastic process with a different initial condition. In this case the input parameter *X0* will not be used.
11. **P0** is $M \times N$ matrix of numeric values where M is the number of compartments and N is the number of the nodes. If the input *drawfromprobdis* is set to *TRUE*, the function interprets the *nth* column of the input *P0* as the probability distribution of initially finding node n in different states. Hence, in order to assign the node n an initial state the function draws a state using the probability distribution defined by the *nth* columns of *P0* or *P0[,n]*

Output Argument The output of *GEMF_SIM_Prob* function is a list that contains a numeric vector, *Tp*, and a numeric three dimensional array ,*compcu*. *lst4=list(Tp,compcu)*.

1. **Tp** is a sequence of time-points, from *t=0* to *t=Runtime* with the step-size equals to the input parameter *timstp*. The function counts the occasions that a node occupies a compartment at a certain time point specified by the elements of Tp.
2. **compcu** The function generates as many realizations of the spreading process as the value of the input parameter *numrun*. The element *compcu[i,n,j]* stores the number of times among all the realizations that the node n occupies the compartment *comp[i]*[3] at the time instant *Tp[j]*. Hence the probability of finding node n in *comp[i]* at the time instant *Tp[j]* can be estimated by the value of *compcu[i,n,j]/numrun*.

- ## Net_Import

Net=Net_Import(File,N)
Generates the *Net* parameter for a single layer topology. *Net* is the input parameter of *GEMF_SIM* function.

Input Arguments

1. **File** is the name of a text file that describes a single layer network.The network links can be directed and weighted. The text file should have three columns where the elements in each row are tab delimited. Each row represents a directed link from the first element in the row to the second element and the weight of the link is the third element. If a link between the nodes i and j is undirected, where the nodes on either side of the link can affect each other, two rows should be included in the text file; One row for describing a link from node i to node j and another row for a link from node j to node i. Hence The corresponding rows would be:
i j 1
j i 1
where the *1* in the third column is the weight of the link.
2. **N** is the number of nodes in the network.

Output Arguments

1. **Net** is a *Net* parameter with *l=1*. The *Net* parameter is described as one of the input arguments for the *GEMF_SIM* function.

[3] *comp* is the input parameter of *GEMF_SIM_Prob* function

- **NetCmbn**

Net=NetCmbn(NetSet,N)
This function combines the *Net* arguments of single-layer networks and generates the *Net* argument for the multilayer network which can be used as the input argument for the *GEMF_SIM* function.

Input Arguments

1. **NetSet** is a list containing the *Net* parameters of single-layer networks. For example if we have two layers then *NetSet=list(Net1,Net2)* where *Net1* and *Net2* are the *Net* parameters of two single-layer networks. Each single layer *Net* parameter can be generated using the *Net_Import* function.
2. **N** is the number of nodes in the network.

Output Arguments

1. **Net** is the *Net* parameter for the multilayer network and can be used as the input argument for the *GEMF_SIM* function.

- **Para_SIS**

Para=Para_SIS(delta,beta)
The argument *Para* for a generic spreading model is described as one of the input arguments of *GEMF_SIM* function. The function *Para_SIS* generates the argument *Para* for the *SIS* spreading model over a single-layer network.

Input Arguments

1. **delta**
2. **beta**
Here *delta* and *beta* are the transition rates in the *SIS* model where each node is *susceptible* or *infected*. In this model a *susceptible* node becomes *infected* with the rate *beta* if it has one *infected* neighbor and an *infected* node becomes *susceptible* with the rate *delta*.

Output Arguments

1. **Para** is the specific *Para* argument for the *SIS* spreading model over a single-layer network. *Para=list(M,q,l,A_d,A_b)*, For the *SIS* spreading model over a single layer network we have
   - number of node states *M=2* (susceptible state is represented by the integer number *1* and the infected state is represented by the integer number *2*)
   - NUMBER OF LAYERS *l=1*
   - INFLUENCER STATES *q=*$[2]$ (because the network is single layer, *q* is 1×1 matrix with the integer number *2* as the influencer state; *2* is representing the *infected* state)
   - NODAL BASED TRANSITION MATRIX $A\_d = \begin{bmatrix} 0 & 0 \\ delta & 0 \end{bmatrix}$ (the only nodal based transition in the *SIS* model is the transition from the *infected* state (represented by *2*) to the *susceptible* state (represented by *1*) with the rate *delta*).
   - EDGE BASED TRANSITION ARRAY *A_b* which has $2 \times 2 \times 1$ ($M \times M \times l$) dimensions and $A\_b[:,:,1] = \begin{bmatrix} 0 & beta \\ 0 & 0 \end{bmatrix}$ (the only edge based transition in the *SIS* model is the transition from the *susceptible* state (represented by *1*) to the *infected* state (represented by *2*) with the rate *beta*).

- **Para_SAIS_2layer**

Para=Para_SAIS_2layer(delta,beta,beta_a,kappa,mu)
The argument *Para* for a generic spreading model is described as one of the input arguments of *GEMF_SIM* function. The function *Para_SAIS_2layer* generates the argument *Para* for the *SAIS* spreading model over a 2-layer network.

Input Arguments

1. **delta**
2. **beta**
3. **beta_a**
4. **kappa**
5. **mu**
   In the *SAIS_2layer* epidemic model each node is either *susceptible* or *infected* or *alert.* If a node is *infected* it becomes *susceptible* with the rate *delta.* If a node is *susceptible* and through the first layer of the network is connected to an *infected* node it becomes *infected* with the rate *beta* or it becomes *alert* with rate *kappa.* Moreover, an *alert* node that has an *infected* neighbor in the first layer becomes *infected* with the rate *beta_a.* In addition to the mentioned transitions, If a node is *susceptible* and has an *infected* neighbor in the second layer of the network, it becomes *alert* with the rate *mu.*

Output Arguments

1. **Para** is the specific *Para* argument for the *SAIS* spreading model over a 2-layer network. *Para=list(M,q,l,A_d,A_b)*, For the *SAIS_2layer* spreading model we have
   - number of node states *M=3* (susceptible state is represented by the integer number *1*, infected state is represented by the integer number *2* and *alert* is represented by the integer number *3*)
   - NUMBER OF LAYERS *l=2*
   - INFLUENCER STATES $q=\begin{bmatrix}2 & 2\end{bmatrix}$ (because the network is 2-layer, q is 1×2 matrix with the integer number *2* as the influencer state; *2* is representing the *infected* state)
   - NODAL BASED TRANSITION MATRIX $A\_d = \begin{bmatrix} 0 & 0 & 0 \\ delta & 0 & 0 \\ 0 & 0 & 0 \end{bmatrix}$ (the only nodal based transition in the *SAIS_2layer* model is the transition from *infected* state to *susceptible* state (*2* → *1*) with the rate *delta*).
   - EDGE BASED TRANSITION ARRAY *A_b* which has $3 \times 3 \times 2$ ($M \times M \times l$) dimensions.
     For the rates of the transitions induced through the first layer of the network we have
     $A\_b[:,:,1] = \begin{bmatrix} 0 & beta & kappa \\ 0 & 0 & 0 \\ 0 & beta\_a & 0 \end{bmatrix}$, which corresponds to the edge based transitions **a.** From *susceptible* state to *infected* state (*1* → *2*) with the rate *beta* **b.** From *susceptible* state to *alert* state (*1* → *3*) with the rate *kappa* **c.** From *alert* state to the *infected* state (*3* → *2*) with the rate *beta_a.*
     For the rates of the transitions induced through the second layer of the network we can write
     $A\_b[:,:,2] = \begin{bmatrix} 0 & 0 & mu \\ 0 & 0 & 0 \\ 0 & 0 & 0 \end{bmatrix}$, which corresponds to the edge based transition from *susceptible* state to *alert* state (*1* → *3*) with the rate *mu.*

• NetGen_Geo

Net=NetGen_Geo(N,r)
This function generates random geometric graph by distributing the nodes uniformly and independently in the unit square and connecting any two nodes by a link if and only if their distance is smaller than a certain value. The function outputs the *Net* parameter for the undirected geometric graph network.

Input Arguments

1. **N** is number of the nodes in the network.
2. **r** In the random geometric graph any pairs of nodes are connected if and only if the distance between them is smaller than *r.*

Output Arguments

1. **Net** is the *Net* parameter for the undirected random geometric graph and it can be fed in to the *GEMF_SIM* function as a input argument.

- **NetGen_ER**

  Net=NetGen_ER(N,p)
  This function generates a variant Erdos-Renyi random graph where any pair of nodes in the network have a fixed probability of being connected. The function outputs the *Net* parameter for the undirected Erdos-Renyi graph.

  Input Arguments

  1. **N** is number of the nodes in the network.
  2. **p** In the Erdos-Renyi random graph any pair of nodes in the graph are independently connected with probability *p*.

  Output Arguments

  1. **Net** is the *Net* parameter for the undirected Erdos-Renyi random graph and it can be fed in to the *GEMF_SIM* function as a input argument.

Examples

- **SIS epidemic over 1-layer network**
  In this example the goal is to generate one realization of the the SIS epidemic model over a single layer network. Here we explain the code that we need to run in R:
  first we start with loading some functions that we will use in the simulation

```
source("NeighborhoodDataWD.R");
source("Net_Import.R");
source("Para_SIS.R");
source("Post_Population.R");
source("GEMF_SIM.R")
```

the next step is to prepare the input arguments for the GEMF_SIM function. The characteristics of the input arguments and the functions are explained in the "Description" section of the manual.

```
File="edgewd.txt";          importing a Net from a file
N=379;
Net=Net_Import(File,N);
Para=Para_SIS(1,0.2);          setting up an SIS epidemic model
x0=matrix(2,1,N);          generating an initial condition where all the nodes are initially infected
maxNumevent=35000;          values that specifies when the simulation terminates
Runtime=30;
```

now the input arguments are defined and we can run the GEMF_SIM function

```
lst=GEMF_SIM(Para,Net,x0,maxNumevent,Runtime,N);
ts=lst[[1]];
n_index=lst[[2]];
i_index=lst[[3]];
j_index=lst[[4]];
Tf=lst[[5]];
lasteventnumber=lst[[6]];
```

using the Post_Population function we can find the population of the compartments through time

```
M=Para[[1]];
lst2=Post_Population(x0,M,N,ts,i_index,j_index,lasteventnumber);
T=lst2[[1]];
StateCount=lst2[[2]];
infectedpopulation=StateCount[2,];
susceptiblepopulation=StateCount[1,];
```

we can plot the population of the infected nodes or the susceptible nodes respect to time

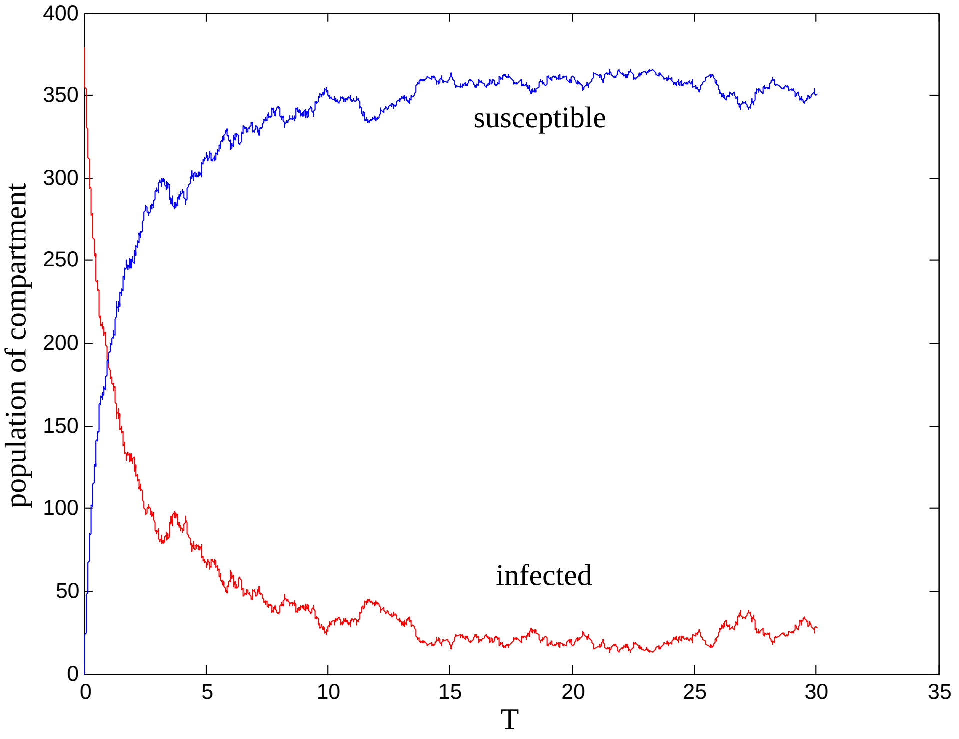

- **SAIS epidemic model over 2-layer network**

In this example we simulate the SAIS epidemic over a 2-layer network and estimate the probability distribution of finding a node in different states as a function of time. For this simulation the initial states of the nodes are chosen from a probability distribution and the network is randomly generated. In order to run the simulation we execute *GEMF_SIM_Prob* function over several cores of the computer in parallel.

first we start with loading some functions that we will use in the simulation

source("NeighborhoodDataWD.R");
source("NetCmbn.R");
source("Para_SAIS_2layer.R");
source("GEMF_SIM_Prob.R");
source("NetGen_Geo.R");
source("NetGen_ER.R");

the next step is to prepare the input arguments for the GEMF_SIM_Prob function. The characteristics of the input arguments and the functions are explained in the "Description" section of the manual.

N=300; *generating a 2-layer random network with 300 nodes*
Net1=NetGen_Geo(N,0.1)
Net2=NetGen_ER(N,0.03);
NetSet=list(Net1,Net2);
Net=NetCmbn(NetSet,N);
Para=Para_SAIS_2layer(1,0.2,0.1,0,0.1); *setting up an SAIS_2layer epidemic model*
M=Para[[1]];
P0=matrix(0,M,N); *generating an initial condition where for each node the probabilities of being*
P0[1,]=0.25; *susceptible, infected or alert, initially are 0.25, 0.5 and 0.25*
P0[2,]=0.5;
P0[3,]=0.25;
maxNumevent=100000; *values that specifies when the simulation terminates*
Runtime=5;
numrun=25; *each core of the computer will make 25 realization of the epidemic process*
comp=c(1,2,3);

we can run GEMF_SIM_Prob function in parallel on several cores

library(parallel);
numcor=4; *here we choose 4 cores to run the simulation*
cl=makeCluster(numcor);

the cluster function makes the list "result" that has the output of GEMF_SIM_Prob, from different cores, as its elements

```
timstp=0.1;
result=clusterCall(cl, GEMF_SIM_Prob, Para,Net,X0=NA,maxNumevent,Runtime,N,numrun,timstp,comp,
drawfromprobdis=TRUE,P0);
stopCluster(cl);
```

we can incorporate the simulations from different cores

```
Tp=result[[1]][[1]];
compcu=result[[1]][[2]];
for (j in 2:numcor){compcu=compcu+result[[j]][[2]]};
s=numcor*numrun;
```

total number of the realization for the epidemic process

```
comppr=compcu/s;
susceptible=colSums(comppr[1,,])
infected=colSums(comppr[2,,])
alert=colSums(comppr[3,,])
```

we can plot the average number of the susceptible, infected or alert nodes respect to time

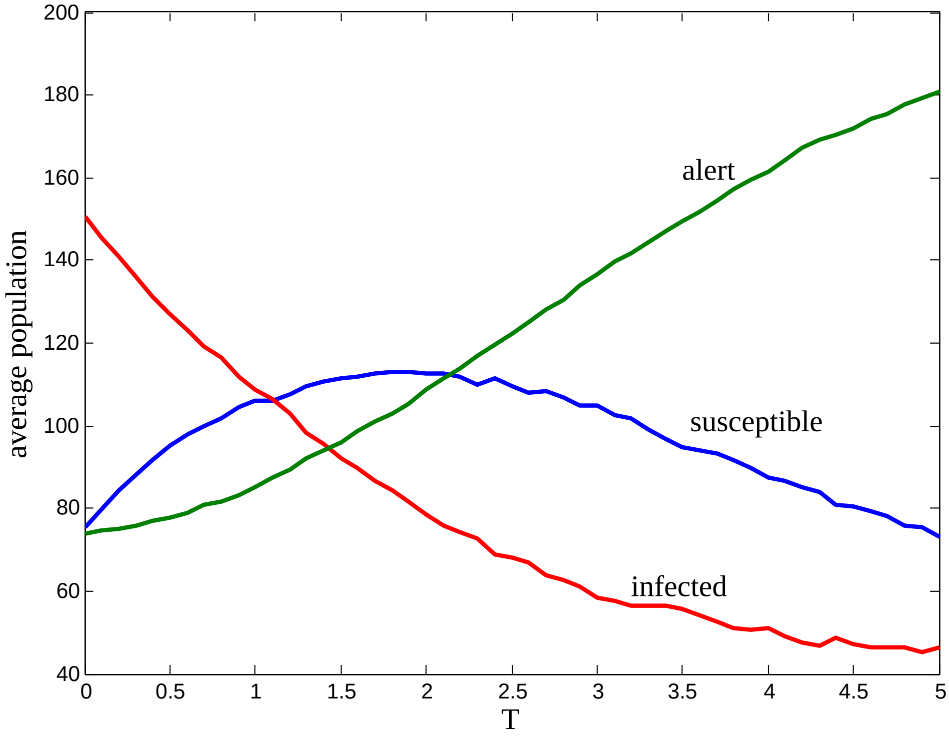

Supplement: Supplementary file 2 — Supporting code and data [file 41598_2019_52501_MOESM2_ESM.zip › Supporting code data/Rtutorial.pdf]
